# Microbiome Analysis Reveals Diversity and Function of *Mollicutes* Associated with the Eastern Oyster, *Crassostrea virginica*

**DOI:** 10.1128/mSphere.00227-21

**Published:** 2021-05-12

**Authors:** Zachary T. Pimentel, Keith Dufault-Thompson, Kayla T. Russo, Abigail K. Scro, Roxanna M. Smolowitz, Marta Gomez-Chiarri, Ying Zhang

**Affiliations:** a Department of Cell and Molecular Biology, University of Rhode Island, Kingston, Rhode Island, USA; b Aquatic Diagnostic Laboratory, Roger Williams University, Bristol, Rhode Island, USA; c Department of Fisheries, Animal and Veterinary Science, University of Rhode Island, Kingston, Rhode Island, USA; Clemson University

**Keywords:** *Chlamydiae*, *Mollicutes*, *Spirochaetia*, eastern oyster, metagenomics, microbiome

## Abstract

Marine invertebrate microbiomes play important roles in diverse host and ecological processes. However, a mechanistic understanding of host-microbe interactions is currently available for a small number of model organisms. Here, an integrated taxonomic and functional analysis of the microbiome of the eastern oyster, Crassostrea virginica, was performed using 16S rRNA gene-based amplicon profiling, shotgun metagenomics, and genome-scale metabolic reconstruction. Relatively high variability of the microbiome was observed across individual oysters and among different tissue types. Specifically, a significantly higher alpha diversity was observed in the inner shell than in the gut, gill, mantle, and pallial fluid samples, and a distinct microbiome composition was revealed in the gut compared to other tissues examined in this study. Targeted metagenomic sequencing of the gut microbiota led to further characterization of a dominant bacterial taxon, the class *Mollicutes*, which was captured by the reconstruction of a metagenome-assembled genome (MAG). Genome-scale metabolic reconstruction of the oyster *Mollicutes* MAG revealed a reduced set of metabolic functions and a high reliance on the uptake of host-derived nutrients. A chitin degradation and an arginine deiminase pathway were unique to the MAG compared to closely related genomes of *Mollicutes* isolates, indicating distinct mechanisms of carbon and energy acquisition by the oyster-associated *Mollicutes*. A systematic reanalysis of public eastern oyster-derived microbiome data revealed a high prevalence of the *Mollicutes* among adult oyster guts and a significantly lower relative abundance of the *Mollicutes* in oyster larvae and adult oyster biodeposits.

**IMPORTANCE** Despite their biological and ecological significance, a mechanistic characterization of microbiome function is frequently missing from many nonmodel marine invertebrates. As an initial step toward filling this gap for the eastern oyster, *Crassostrea virginica*, this study provides an integrated taxonomic and functional analysis of the oyster microbiome using samples from a coastal salt pond in August 2017. The study identified high variability of the microbiome across tissue types and among individual oysters, with some dominant taxa showing higher relative abundance in specific tissues. A high prevalence of *Mollicutes* in the adult oyster gut was revealed by comparative analysis of the gut, biodeposit, and larva microbiomes. Phylogenomic analysis and metabolic reconstruction suggested the oyster-associated *Mollicutes* is closely related but functionally distinct from *Mollicutes* isolated from other marine invertebrates. To the best of our knowledge, this study represents the first metagenomics-derived functional inference of *Mollicutes* in the eastern oyster microbiome.

## Abstract (precis)

Despite their biological and ecological significance, a mechanistic characterization of microbiome function is frequently missing from many nonmodel marine invertebrates. As an initial step toward filling this gap for the eastern oyster, *Crassostrea virginica*, this study provides an integrated taxonomic and functional analysis of the oyster microbiome using samples from a coastal salt pond in August 2017.

## INTRODUCTION

Oysters are filter-feeding bivalve molluscs with great ecological and economic significance. They are considered ecosystem engineers due to their ability to form reefs that serve a variety of beneficial functions, including protecting shorelines from storm-related damage and providing habitat for other marine organisms (1, 2). Eastern oysters have been shown to play roles in coastal biogeochemical cycles, for example, through promoting denitrification and accumulation of heavy metals (3–5). For these reasons, efforts have been made to restore oyster populations around the United States. In addition, oysters represent a significant and growing portion of the aquaculture industry. In 2018, *Crassostrea* spp. of oysters represented almost one-third of the major species produced in world aquaculture (6). Given their ecological and economic importance, a more comprehensive understanding of the factors that mediate oyster physiology would have potential implications in coastal management and the aquaculture industry.

Like other invertebrates, oysters are known to harbor a diverse range of microorganisms. Culture-based studies have revealed the presence of *Proteobacteria* (e.g., *Vibrio*, Pseudomonas, *Alteromonas*), *Actinobacteria* (e.g., *Micrococcus*), and *Bacteroidetes* (e.g., *Flavobacterium*) in the oyster-associated microbiome (7). Some readily culturable members, such as strains of the *Vibrio* genus, have been closely studied to profile their abundance (8), pathogenic potential (9), evolution and diversity (10), and inhibitions by probiotics (11, 12). Other well-known microbes from the eastern oyster include several protozoan pathogens, such as Perkinsus marinus, Haplosporidium nelsoni, and Haplosporidium costale, causative agents of Dermo, Multinucleated Sphere X (MSX), and Seaside Organism (SSO) diseases, respectively, which are major diseases of adult eastern oysters (13). However, less is known about nonculturable microbes and their potential associations with diverse physiological and ecological functions of eastern oysters (14, 15).

Culture-independent approaches, such as the profiling of amplicon libraries, have led to the detection of other previously uncultured taxa in the oyster microbiome, such as the *Chloroflexi*, *Firmicutes*, *Fusobacteria*, *Planctomycetes*, *Spirochaetes*, *Tenericutes*, and *Verrucomicrobia* (16, 17). A distinct microbiome is found in multiple oyster tissues (e.g., hemolymph, gill, mantle, and gut) compared with microbial communities of the surrounding seawater, suggesting potential host selection strategies that lead to the enrichment of specific groups (18). Multiple factors, including changes in environmental conditions (16, 19–21), diet (22), infection (23, 24), and the use of probiotics (25), have been shown to influence the composition of oyster microbiomes during certain life stages and among different tissue types. All of these studies set the stage for further investigating the taxonomic composition and functional potential of oyster microbiomes across different tissues.

In the absence of microbial isolates, shotgun metagenomics serves as a useful tool for gaining functional insights into uncultured members of a microbiome. Prior applications of metagenomics in marine invertebrates have revealed remarkable bacterial functions, including chemical defense mediated by secondary metabolites produced by the sponge microbiome (26) and the metabolic interactions between chemosynthetic symbionts and their hosts in deep-sea hydrothermal vents (27). One potential challenge in the application of metagenomics to host tissues is the high abundance of host DNA that masks the signals from the tissue-associated microbiome (28). This issue may not be easily resolved by targeting different sample types (e.g., using biodeposit samples to represent the gut microbiota), as a clear distinction is found between the fecal microbiome and the gut microbiome of filter-feeding bivalves, e.g., the blue mussel, Mytilus edulis (29). Thus, a successful application of metagenomics in oyster microbiome studies requires the development of customized protocols for the enrichment of microbial DNA from the host tissue.

Here, an integrated microbiome analysis of the eastern oyster was performed by combining 16S rRNA gene-based community profiling, shotgun metagenomics, and genome-scale metabolic reconstruction. Amplicon libraries were analyzed to compare the diversity and distribution of microbiomes across six distinct oyster tissues and between oysters infected and uninfected with the protozoan pathogen *P. marinus*. Metagenomic-based identification of oyster gut-associated bacteria was enabled with a specialized protocol that enriched microbes from host tissues. Functional characterization was performed through the application of a genome-scale metabolic reconstruction on a metagenome-assembled genome (MAG). Together, these analyses shed light on the diversity of the eastern oyster microbiome across tissue types and provided functional insights into the *Mollicutes*, a prevalent taxon in the microbiome of the eastern oyster.

## RESULTS

### Sample descriptions.

Tissue samples were dissected from 29 oysters collected from an aquaculture farm in Ninigret Pond, Rhode Island, USA. The 16S rRNA gene-based amplicon sequencing was performed on multiple tissues of 19 oysters, and a microbiome-enriched metagenome was sequenced on the gut samples pooled from the remaining 10 oysters (see Data Set S1A in the supplemental material). Of the 19 oysters, amplicon samples from the pallial fluid and hemolymph were collected from 9 oysters, amplicon samples from the gut, mantle, gill, and inner shell were collected from all, and additional metagenome samples were prepared from the gut of 12 oysters (Materials and Methods). Individual oysters were tested for potential infection by common causative agents of oyster disease in the region, including *P. marinus* (causative agent of Dermo disease), *H. nelsoni* (causative agent of MSX disease), and *H. costale* (causative agent of SSO disease) (Materials and Methods). Overall, 14 of the 29 oysters were infected with *P. marinus* with a range of infection severities from 0.5 to 5 as measured by the Mackin index (30, 31), while little to no infection from *H. nelsoni* or *H. costale* was detected among all 29 samples (Data Set S1A). Due to the low number of individuals infected with *H. nelsoni* and *H. costale*, these infections were not considered a factor in subsequent analyses.

10.1128/mSphere.00227-21.6 DATA SET S1 Data associated with the 16S rRNA gene-based community profiling analysis. (A) Summary of the oyster samples analyzed in this study, including the mass, size, protozoan pathogen quantification, and sample and library information related to the amplicon and metagenomic sequencing. Oysters used for amplicon sequencing were marked with “X” in the columns “Amplicon Sequencing” and “Hemolymph and Pallial Fluid amplicon sequencing.” The 12 metagenomic samples from round 1 of shotgun metagenomic sequencing were marked with their corresponding sample identifiers (ZTP1 to ZTP12) in the column “Individual Gut Metagenome Sequenced (Round I),” and the pool of oysters used for preparing the microbiome-enriched metagenomic sample was marked with “X” in the column “Gut Included in the Pooled Metagenomic Sequencing (Round II).” The number of reads obtained from each amplicon sequencing library was shown, with a sample marked with yellow background removed from further analysis due to low sequencing depth. (B) Summary of ASV counts and the percentage of conserved ASVs by tissue type. (C) Statistical significance in the comparison of community profiles between tissues. Statistically significant differences were marked with a yellow background (*P* value < 0.05). (D) Relative abundance of the main taxa in oyster microbiomes across different tissue types. Log fold change and statistical significance were estimated using the ANCOM-BC approach. Download Data Set S1, XLSX file, 0.03 MB. Copyright © 2021 Pimentel et al. 2021 Pimentel et al. https://creativecommons.org/licenses/by/4.0/ This content is distributed under the terms of the Creative Commons Attribution 4.0 International license.

### Community diversity of the oyster microbiome.

**FIG 1:**
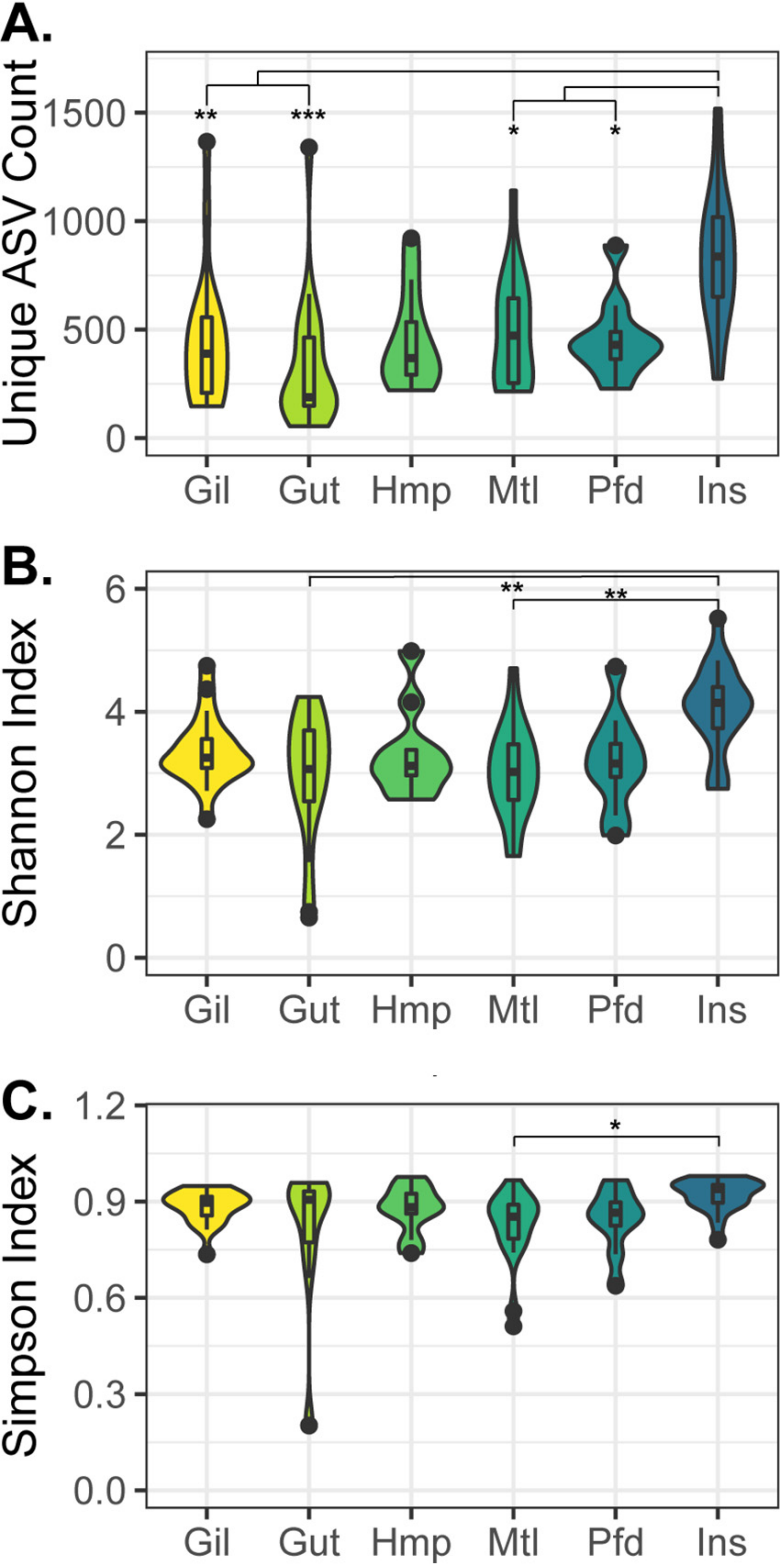
Diversity of eastern oyster microbiome samples as measured by unique ASV count (A), Shannon index (B), and Simpson index (C). Samples were grouped by tissue type, with abbreviations as follows: Gil (gill), Gut (gut), Hmp (hemolymph), Mtl (mantle), Pfd (pallial fluid), and Ins (inner shell). Pairwise statistical significance was assessed using a pairwise Wilcoxon test with Holm *P* value adjustment for multiple comparisons: *, *P* value < 0.05; **, *P* value < 0.01; ***, *P* value < 0.001.

The alpha diversity of the oyster microbiome was compared among different tissue types using the unique amplicon sequence variant (ASV) counts, the Shannon index, and the Simpson index across all the profiled oyster samples (Fig. 1). The unique ASV counts revealed a significantly higher diversity in the inner shell samples than in the gut, gill, mantle, and pallial fluid samples (*P* value < 0.05, Wilcoxon test); the Shannon index revealed a higher diversity in the inner shell than the gut and mantle (*P* value < 0.05, Wilcoxon test); and the Simpson index showed statistically significant difference only between the inner shell and the mantle (*P* value < 0.05, Wilcoxon test). A high level of heterogeneity was observed among the different tissues, with the median number of unique ASVs per sample ranging from 188 in the gut to 838 in the inner shell (Data Set S1B). Similarly, when comparing the pooled ASVs from different oyster tissues, a total of 15,011 unique ASVs were identified among all the oyster samples, but only 496 ASVs (3.3%) were present in all six tissue types analyzed (Fig. S1). A high level of heterogeneity was also observed among individual oysters even when the same tissue type was examined, with the gut and mantle representing the lowest percentage (0.6%) and the pallial fluid representing the highest percentage (3.4%) of conserved ASVs (present in >80% of samples for a given tissue type) identified from each tissue type (Data Set S1B). Correlations between *P. marinus* infection and microbiome alpha diversity were examined within each tissue type. Significant differences were identified only in the inner shell samples, where the uninfected samples were significantly more diverse than the infected samples (*P* value < 0.05, Wilcoxon test) based on all three alpha diversity measures.

10.1128/mSphere.00227-21.1 FIG S1 Venn diagram showing the overlap of ASVs identified from different oyster tissues. ASVs observed in at least one sample of a tissue type were counted as present in that tissue type. The number of samples in each tissue type can be found in Data Set S1A. Download FIG S1, TIF file, 1.3 MB. Copyright © 2021 Pimentel et al. 2021 Pimentel et al. https://creativecommons.org/licenses/by/4.0/ This content is distributed under the terms of the Creative Commons Attribution 4.0 International license.

The beta diversity among different samples was measured based on phylogenetic isometric log-ratio (PhILR)-transformed ASV counts (Materials and Methods). Statistical analysis of the sample distances suggested that the gut samples were the most distinct of the profiled tissue types, as they were significantly different from all other tissue types (*P* value < 0.05, pairwise permutational multivariate analysis of variance [PERMANOVA]). The gill, mantle, and inner shell samples were also significantly different from one another, while the pallial fluid and hemolymph samples had no statistically significant difference from other tissue types with the exception of the gut (Data Set S1C). The comparison of *P. marinus*-infected against uninfected samples within each tissue type revealed no statistical significance in beta diversity (*P* value < 0.05, PERMANOVA).

### Taxonomic abundance among oyster tissues.

**FIG 2:**
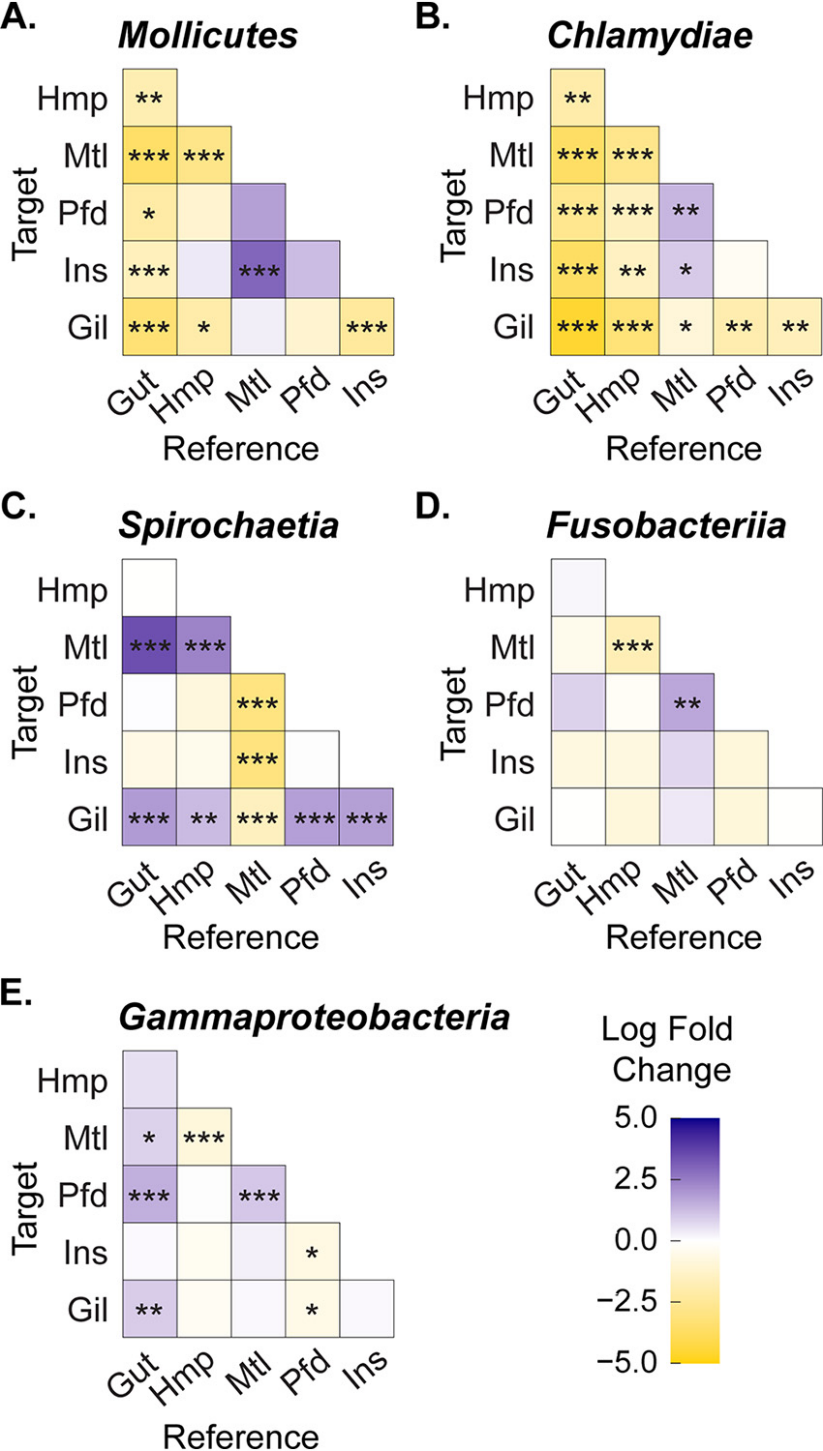
Heatmap showing the log fold change (computed with ANCOM-BC) of major oyster microbiome taxa across different tissue samples: *Mollicutes* (A), *Chlamydiae* (B), *Spirochaetia* (C), *Fusobacteriia* (D), and *Gammaproteobacteria* (E). Each tissue was assigned an abbreviation as follows: Gil (gill), Gut (gut), Hmp (hemolymph), Mtl (mantle), Pfd (pallial fluid), and Ins (inner shell). Tissue labels representing rows (on the left-hand side of each heatmap) are the target tissue while the labels representing columns (on the bottom of each heatmap) are the reference tissue. For example, a significantly lower relative abundance of the *Mollicutes* was observed in the hemolymph compared to the gut samples. Pairwise statistical significance was assessed with ANCOM-BC: *, *P* value < 0.05; **, *P* value < 0.01; ***, *P* value < 0.001.

Taxonomic assignment of the ASVs obtained from this study revealed several major bacterial classes that had a median relative abundance of greater than 10% within at least one tissue type, including *Mollicutes*, *Chlamydiae*, *Spirochaetia*, *Fusobacteriia*, and *Gammaproteobacteria* (Fig. 2). The relative abundance of these bacterial classes was examined among different tissue types using the ANCOM-BC (analysis of compositions of microbiomes with bias correction) approach (32). The gut samples had a significantly higher relative abundance of *Mollicutes* and *Chlamydiae* than any other tissue types examined, with an estimated log fold change ranging from 1.6 to 3.6 for the *Mollicutes* and from 2.0 to 4.6 for the *Chlamydiae* compared to the other tissues (Fig. 2A and B; Data Set S1D). Similarly, the mantle and gill samples had a significantly higher abundance of *Spirochaetia* than other tissues, with the mantle having 2- to 3-fold-higher abundance than other tissue types and 1.5-fold-higher abundance than the gill (Fig. 2C; Data Set S1D). The hemolymph had around a 2-fold-higher relative abundance of *Mollicutes*, *Chlamydiae*, and *Fusobacteriia* compared to the mantle (Fig. 2A, B, and D; Data Set S1D). The gill had a significantly lower abundance of *Chlamydiae* compared to all other tissue types, while the inner shell had a significantly higher abundance of *Mollicutes* than the mantle and gill and a lower abundance of *Gammaproteobacteria* than the pallial fluid samples (Fig. 2A and E).

### Metagenomic sequencing of the oyster gut microbiome.

The shotgun metagenomic sequencing of oyster gut microbiomes resulted in the collection of over 2.5 billion raw reads and 2.1 billion quality-filtered reads. Around 1.7 billion (80%) and 7.8 million (0.4%) of the quality-filtered reads were assigned to the oyster host and *P. marinus*, respectively. A total of 394,986,464 reads that were unmapped to the eastern oyster or *P. marinus* genomes were coassembled across the two rounds of metagenomic sequencing, resulting in 612,574 unique contigs. At least one nearly complete 16S rRNA gene was detected for each of the five major bacterial classes (along with the *Bacteroidetes* and *Cyanobacteria*), ranging from 1,396 to 1,562 bp in length. Six of the full-length 16S rRNA genes were mapped to the amplicon data with full coverage and 100% identity to the corresponding ASVs. These ASV-mapped full-length 16S rRNA genes were taxonomically assigned to the *Mollicutes* (named *Mollicutes-1* and *Mollicutes-2*), *Chlamydiae*, *Spirochaetia* (named *Spirochaetia-1* and *Spirochaetia-2*), and *Bacteroidia* (Data Set S2A).

**FIG 3:**
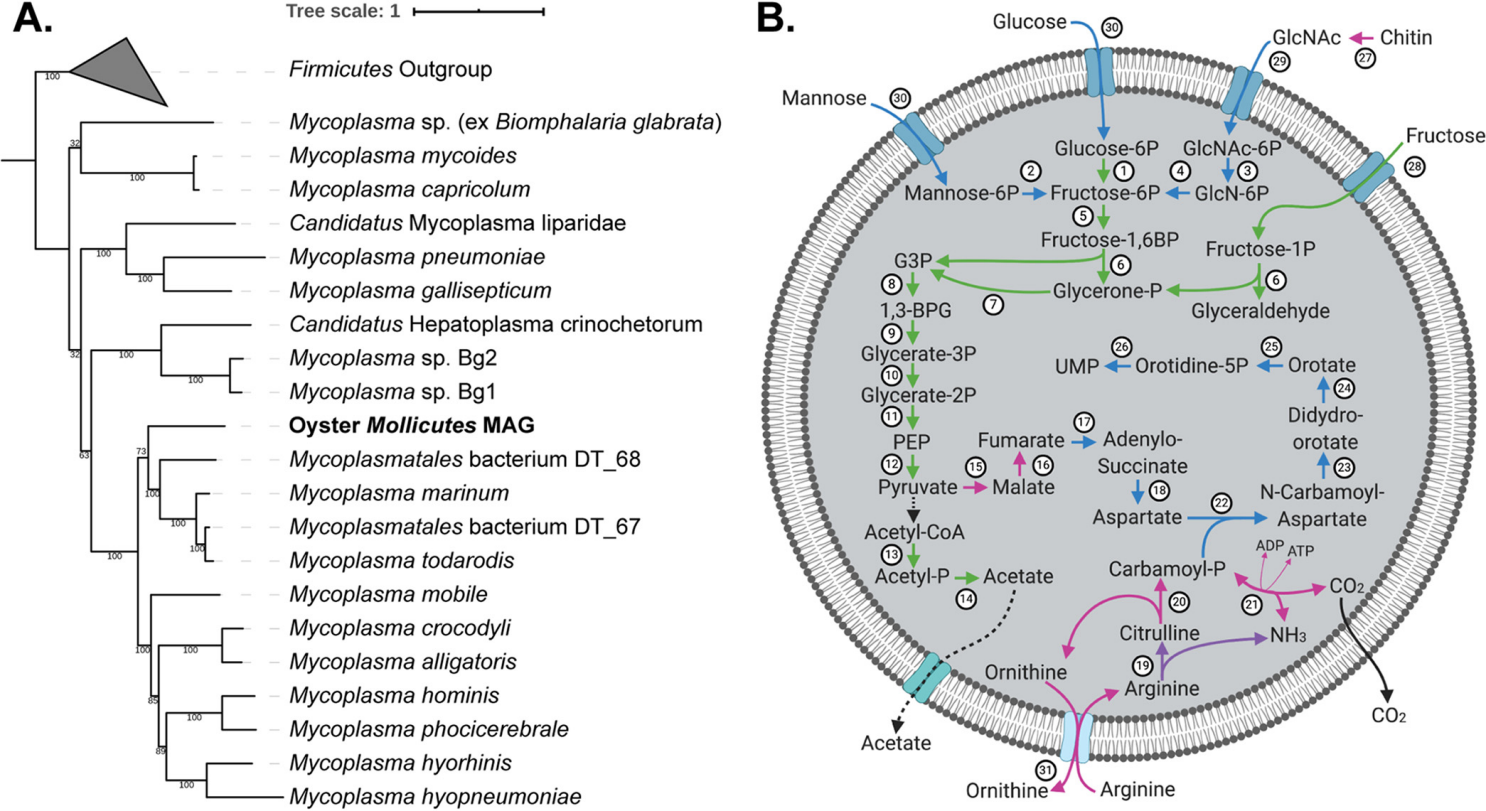
Characterization of a metagenome-assembled genome (MAG) of the *Mollicutes* from the oyster microbiome. (A) Maximum likelihood phylogenomic reconstruction based on conserved single-copy genes. Members of the *Firmicutes* were selected as outgroups in the phylogeny (Data Set S2B). Support values were based on 100 iterations of bootstrapping. (B) Visualization of the central metabolic pathways reconstructed from the oyster *Mollicutes* MAG (created with BioRender). Metabolites are connected with directed edges indicating biochemical conversions or transport and diffusion processes. The edges were coded by circled numbers, with further details represented in Data Set S2D. Additional coding was provided by the edge colors to indicate conservations between the oyster *Mollicutes* MAG and reference genomes, including the marine host-associated *Mycoplasma marinum* and *Mycoplasma todarodis* and the freshwater host-associated Mycoplasma mobile. Green, conserved in all four genomes; blue, conserved between the *Mollicutes* MAG and the marine *Mycoplasma* (M. marinum and *M. todarodis*) but absent in *M. mobile*; purple, conserved between the *Mollicutes* MAG and the freshwater *M. mobile* but absent from the marine *Mycoplasma*; magenta, unique functions in the *Mollicutes* MAG. The conversion from pyruvate to acetyl-CoA was marked as black because the function of a pyruvate dehydrogenase was identified outside the MAG from unbinned contigs that had top BLAST hits to members of the *Mycoplasma*.

**FIG 4:**
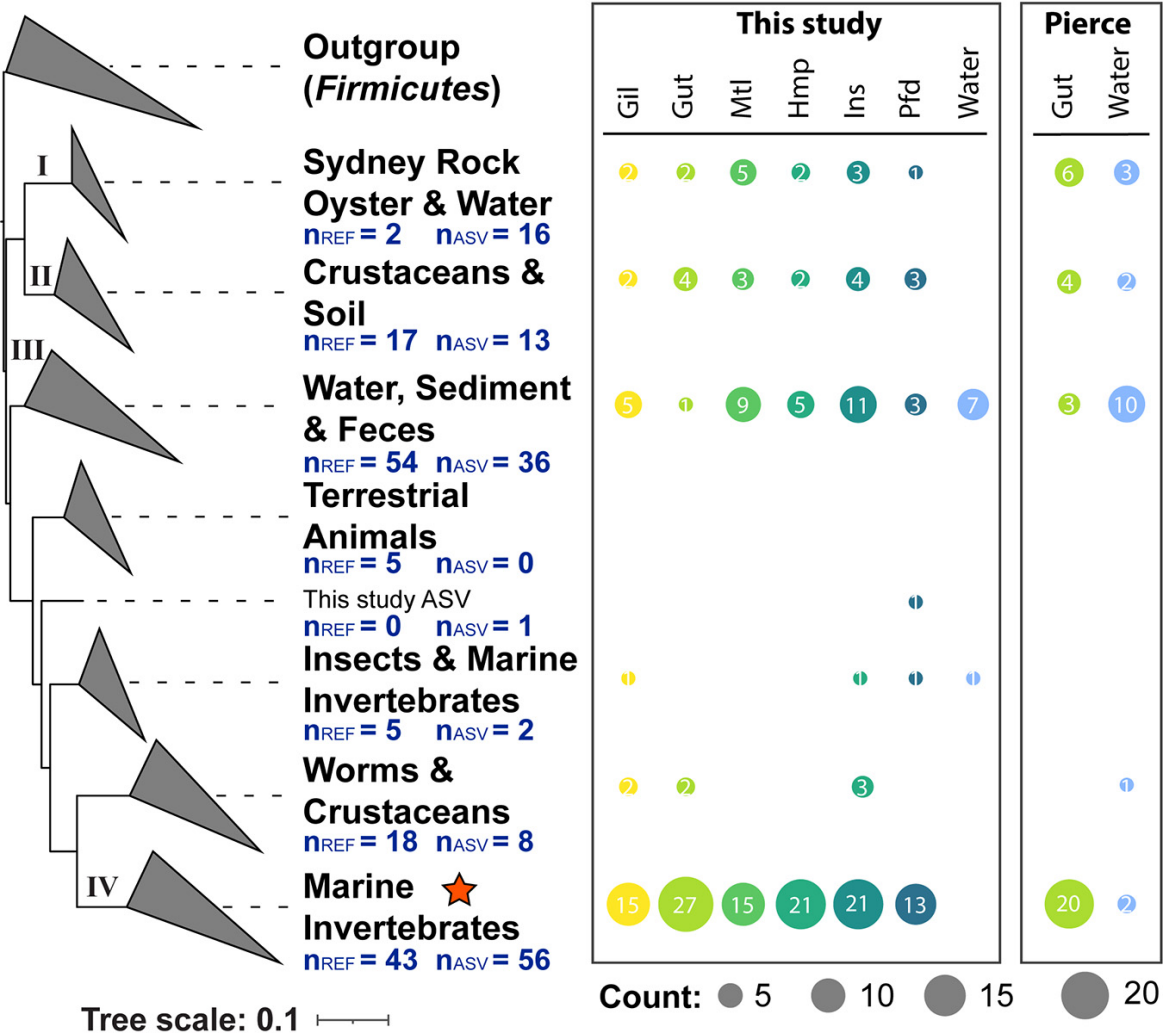
Phylogenetic positioning of oyster- and water-associated *Mollicutes* ASVs. The branches were collapsed to summarize the environments represented by reference 16S rRNA genes in the SILVA database. The four major clades of oyster-associated *Mollicutes* were labeled with Roman numerals I to IV. The number of SILVA reference sequences (n_REF_) and unique ASV sequences (n_ASV_) were marked in each collapsed clade. Corresponding counts of unique ASVs in the different sample types were shown as a bubble plot for each clade on the right of the phylogeny, with filled circles sized according to the ASV counts and colored based on sample types. Data from this study and a prior study (39) were included in the analysis. Sample types included water and the following oyster tissues: Gil (gill), Gut (gut), Hmp (hemolymph), Mtl (mantle), Pfd (pallial fluid), and Ins (inner shell). A given ASV may appear in multiple sample types, and thus, the sum of values in each row of the bubble plot is not necessarily the number of ASVs (n_ASV_) in the collapsed branch. The orange star indicates the clade where the metagenome-assembled full-length 16S rRNA genes, *Mollicutes-1* and *Mollicutes-2*, and their corresponding ASVs are located (Data Set S2E).

10.1128/mSphere.00227-21.7 DATA SET S2 Data associated with the metagenomics analysis. (A) Metagenome-assembled full-length 16S rRNA genes. Sequences with 100% identity and full coverage to ASVs identified in this study were shown with the corresponding ASV identifiers. Taxonomic assignments were based on a comparison to reference genes in the SILVA database. (B) Average nucleotide identity (ANI), average amino acid identity (AAI), and conserved gene counts of the oyster *Mollicutes* MAG compared to reference genomes and outgroups analyzed in the phylogenomic reconstruction (Fig. 3A). (C) Average nucleotide identity (ANI), average amino acid identity (AAI), and conserved gene counts of the oyster *Chlamydiae* MAG compared to reference genomes and outgroups analyzed in the phylogenomic reconstruction (Fig. S3). (D) Metabolic functions corresponding to the visualization of central metabolism reconstructed from the oyster *Mollicutes* MAG. Each number in the Reaction Number column refers to a metabolic function correspondingly labeled in Fig. 3B. Gene identifiers corresponding to each function were included under the four genomes: *M. mobile*, M. marinum, *M. todarodis*, and oyster *Mollicutes* MAG. Each entry was classified in the Type column based on conservation of genes between the *Mollicutes* MAG and other genomes: All4, functions conserved in all four genomes analyzed; MAG+Marine, functions conserved between the MAG and the marine species M. marinum and *M. todarodis*; MAG+Mobile, functions conserved between MAG and *M. mobile*; MAG Only, functions unique to the MAG. Additional information including the reaction name, metabolic pathway(s), Enzyme Commission numbers (EC Number), COG classification, and eggNOG annotations was provided. (E) Metadata showing the references and ASVs included in the phylogeny of Fig. 4. Reference genes were labeled as identifiers in the SILVA reference sequence database. ASVs were labeled as their unique identifiers. The Clade assignments indicate the positioning of a reference or ASV to a specific clade in Fig. 4. More specific information about the source environment of each reference gene was represented in the Environment column. The samples where each ASV was observed (this study or reference 39) were indicated in the Sample Types column. The Taxonomy column indicates the taxonomy assignments for ASVs (based on the QIIME2 sklearn classifier as described in Materials and Methods) and the reference genes (based on taxonomy assignment from SILVA version 138.1). (F) Strain names and NCBI RefSeq assembly accession numbers for a set of manually curated draft genomes of oyster-derived isolates. Download Data Set S2, XLSX file, 0.2 MB. Copyright © 2021 Pimentel et al. 2021 Pimentel et al. https://creativecommons.org/licenses/by/4.0/ This content is distributed under the terms of the Creative Commons Attribution 4.0 International license.

The relative abundance of these 16S rRNA genes was probed across different oyster tissues and between *P. marinus*-infected and uninfected samples using ANCOM-BC and based on mappings to the ASV abundances (Fig. S2). The *Mollicutes-1* showed a significant differential abundance (*P* value < 0.001, ANCOM-BC) between the *P. marinus*-infected and uninfected samples of the gut and the inner shell, where it was more abundant among the uninfected samples of the gut (log fold change of 4.3) but was slightly less abundant in the uninfected samples of the inner shell (log fold change of −1.2). A significantly higher relative abundance of the *Mollicutes-2* (*P* value < 0.001, ANCOM-BC) and *Bacteroidia* (*P* value < 0.05, ANCOM-BC) was also observed in *P. marinus*-uninfected than the infected pallial fluid samples (log fold change of 5.2 and 2.3, respectively). In contrast, the *Chlamydiae* had significantly lower relative abundance in the uninfected gill, mantle, and inner shell samples compared to the *P. marinus*-infected samples (log fold changes of −0.3, −0.2, and −1.1, respectively; *P* value < 0.001, ANCOM-BC).

10.1128/mSphere.00227-21.2 FIG S2 Heatmap showing the differential relative abundance of metagenome-assembled full-length 16S rRNA genes, as measured by their corresponding ASVs, between *Perkinsus marinus*-infected and uninfected tissue samples. Positive log fold change indicates a higher relative abundance of an ASV in *P. marinus*-uninfected samples, while negative log fold change indicates a lower relative abundance of an ASV in *P. marinus*-uninfected samples. Statistical significance was calculated based on ANCOM-BC and marked as follows: *, *P* value < 0.05; **, *P* value < 0.01; ***, *P* value < 0.001. Download FIG S2, TIF file, 0.5 MB. Copyright © 2021 Pimentel et al. 2021 Pimentel et al. https://creativecommons.org/licenses/by/4.0/ This content is distributed under the terms of the Creative Commons Attribution 4.0 International license.

Binning of the coassembled contigs produced two MAGs with completeness greater than 80% and contamination less than 2%. Each MAG contained a full-length 16S rRNA gene, with the first from the class *Mollicutes* (*Mollicutes-1*) and the second from *Chlamydiae*. The *Mollicutes* MAG included 47 contigs with a total length of 0.62 Mb and a GC content of 28.7%. The estimated completeness and contamination were 97.4% and 1.8%, respectively. Similarly, the *Chlamydiae* MAG included 57 contigs with a total length of 1.08 Mb and a GC content of 41.0%, and it had a completeness of 86.2% and contamination of 0.4%. Besides the *Mollicutes* and *Chlamydiae* MAGs, a partial *Spirochetes* MAG was reconstructed, with a completeness of 43.2%, a contamination of 0.7%, and a full-length 16S rRNA gene (*Spirochaetia-1*) included in the MAG.

Phylogenomic reconstructions were performed based on conserved single-copy genes (CSCGs) identified between the two nearly complete oyster MAGs and corresponding reference genomes in the *Mollicutes* and *Chlamydiae* taxa (Materials and Methods). In total, 34 CSCGs and 179 CSCGs were used to build the *Mollicutes* and *Chlamydiae* phylogenies, respectively. Based on the phylogenomic reconstruction, the oyster *Mollicutes* MAG formed a basal branch to a group of marine *Mycoplasma*, with the nearest neighboring branches including Mycoplasma todarodis (isolated from a squid), Mycoplasma marinum (isolated from an octopus), and *Mycoplasmatales* bacterium DT_67 and DT_68, two MAGs obtained from deep-sea sinking particles (herein referred to as DT_67 and DT_68) (33) (Fig. 3A). Meanwhile, the *Chlamydiae* MAG was found within the order *Parachlamydiales* most closely related to Simkania negevensis, an obligate intracellular bacterium with a broad host range from amoebae to animals (34, 35) (Fig. S3).

10.1128/mSphere.00227-21.3 FIG S3 Maximum likelihood phylogenomic reconstruction of the oyster *Chlamydiae* MAG based on conserved single-copy genes identified from the MAG and reference *Chlamydiae* genomes (Data Set S2C). Species of the *Chlamydiales* order were collapsed into a single clade and formed a neighboring clade to the *Parachlamydiales* clade that includes the oyster *Chlamydiae* MAG. Members of the *Verrucomicrobia* were used as outgroups in the phylogeny. Support values were based on 100 iterations of bootstrapping. Download FIG S3, TIF file, 0.5 MB. Copyright © 2021 Pimentel et al. 2021 Pimentel et al. https://creativecommons.org/licenses/by/4.0/ This content is distributed under the terms of the Creative Commons Attribution 4.0 International license.

To determine the similarity of the *Mollicutes* and *Chlamydiae* MAGs to genomes within their corresponding taxa, average nucleotide identity (ANI) and average amino acid identity (AAI) were calculated between the MAGs and all other species represented in their corresponding CSCG phylogenies (Data Set S2B and C). For the *Mollicutes* MAG, the highest pairwise ANI values were observed with M. marinum (66.8%), DT_67 (66.8%), DT_68 (66.4%), and *M. todarodis* (66.0%), all representing strains from marine sources, as well as Mycoplasma mobile (66.1%), a freshwater fish pathogen. Similarly, some of the highest pairwise AAI values were observed with M. marinum (51.9%) and *M. todarodis* (50.3%) with orthologs identified from 57.4% and 53.3%, respectively, of the protein-coding genes in the oyster *Mollicutes* MAG. While DT_67 and DT_68 had slightly higher AAI values of 54.4% and 53.6%, respectively, only a limited number of orthologs were identified, covering 22.5% and 22.2% of the protein-coding genes in the *Mollicutes* MAG, respectively (Data Set S2B). The oyster *Chlamydiae* MAG had an ANI between 62.8% and 64.3% to all reference genomes of the *Chlamydiae* phylum. The highest AAI value (47.9%) was observed with its nearest neighbor in the phylogenomic reconstruction, Simkania negevensis, with ortholog identifications for 53.6% of the protein-coding genes in the *Chlamydiae* MAG (Data Set S2C). Overall, both the *Mollicutes* and *Chlamydiae* MAGs had lower ANI and AAI values to the reference genomes than expected for strains of the same species (both generally ≥95%) (36, 37).

### Novel functional potentials of the oyster *Mollicutes* MAG.

A genome-scale metabolic reconstruction was performed to illustrate the metabolic capacity encoded by the oyster *Mollicutes* MAG (Fig. 3B). Overall, the *Mollicutes* MAG encoded a highly reduced metabolism with few carbon utilization pathways and limited biosynthetic capability. Transport via the phosphotransferase system (PTS) was predicted for glucose, fructose, mannose, and *N*-acetylglucosamine (GlcNAc). A complete glycolysis pathway and all subunits of ATP synthase were present, while genes of the tricarboxylic acid (TCA) cycle were largely missing. The ability to convert pyruvate to fumarate was predicted based on the presence of a malate dehydrogenase and a fumarate hydratase. The conversion of acetyl coenzyme A (acetyl-CoA) to acetate was inferred by the identification of a phosphate acetyltransferase and an acetate kinase. An arginine deiminase (ADI) pathway was identified, potentially contributing to the production of ATP via the exchange of arginine and ornithine (38) and enabling the production of precursors for a *de novo* pyrimidine biosynthesis pathway. The *Mollicutes* MAG also encoded a putative chitinase with the catalytic domain homologous to an experimentally verified chitinase in Pyrococcus furiosus (PDB: 2DSK) and a chitin-binding domain at the C terminus. It was noted that a mechanism of converting pyruvate to acetyl-CoA was missing in the MAG. However, putative subunits of a pyruvate dehydrogenase complex were identified from other contigs in the metagenomic coassembly, with top BLAST hits to members of the *Mycoplasma* genus. Therefore, the potential conversion of pyruvate to acetyl-CoA was speculated in the metabolic reconstruction (Fig. 3B).

A comparative genomic analysis was performed between the oyster *Mollicutes* MAG and genomes from closely related *Mycoplasma* isolates, including two marine species, M. marinum and *M. todarodis*, as well as one freshwater species, *M. mobile* (Data Set S2D). Genes in the oyster *Mollicutes* MAG can be largely classified into four categories based on their conservation with other species from the comparative genomic analysis (Fig. S4). Over 40% of the genes in the *Mollicutes* MAG were conserved among all reference genomes, encoding functions in central metabolism (e.g., ATP synthase, amino acid metabolism, nucleotide metabolism, etc.) and information storage and processing (e.g., replication, translation, transcription). Around 10% of genes were conserved between the *Mollicutes* MAG and the two marine isolates but were missing from *M. mobile*. These included genes belonging to a *de novo* pyrimidine biosynthesis pathway, multiple carbohydrate utilization genes, and one gene that encodes an alpha-amylase domain-containing protein. Only 18 genes (less than 3%) were uniquely conserved between the *Mollicutes* MAG and *M. mobile*, including an arginine deiminase and genes associated with DNA repair and the repair of enzymes under oxidative stress. Finally, around 36% of the genes were unique to the *Mollicutes* MAG. These included genes involved in pyruvate metabolism (e.g., malate dehydrogenase and fumarate hydratase) and the ADI pathway (e.g., carbamate kinase, ornithine carbamoyltransferase, and the arginine/ornithine antiporter). Overall, the oyster *Mollicutes* MAG maintained conserved functions with known *Mycoplasma* species while carrying unique metabolic capability in its genome.

10.1128/mSphere.00227-21.4 FIG S4 Distribution of COG functions among the conserved and unique genes of the oyster *Mollicutes* MAG. The COG functions were profiled among four distinct gene groups based on conservation between the oyster *Mollicutes* MAG and reference genomes, including the marine host-associated *Mycoplasma marinum* and *Mycoplasma todarodis* and the freshwater host-associated Mycoplasma mobile: All 4, conserved in all four genomes; *Mollicutes* MAG Only, unique functions in the *Mollicutes* MAG; *Mollicutes* MAG & Marine Isolates, conserved between the *Mollicutes* MAG and the marine *Mycoplasma* (M. marinum and *M. todarodis*) but absent in *M. mobile*; *Mollicutes* MAG & *M. mobile*, conserved between the *Mollicutes* MAG and the freshwater *M. mobile* but absent from the marine *Mycoplasma*. Download FIG S4, TIF file, 0.5 MB. Copyright © 2021 Pimentel et al. 2021 Pimentel et al. https://creativecommons.org/licenses/by/4.0/ This content is distributed under the terms of the Creative Commons Attribution 4.0 International license.

### Presence and distribution of *Mollicutes* across eastern oyster microbiome studies.

Following functional inferences obtained from the metabolic reconstruction of the oyster *Mollicutes* MAG, emphasis was placed on the composition and diversity of *Mollicutes* across different oyster microbiomes obtained from this and other studies (Fig. S5). A total of three additional data sets were identified from public databases, including the community profiling of gut samples from the adult eastern oyster (39), the profiling of adult eastern oyster biodeposits (40), and the profiling of eastern oyster larvae homogenate (25). Raw data from these prior studies were downloaded and reanalyzed with a standardized pipeline to minimize potential inconsistencies caused by variations in the computational procedures (Materials and Methods). According to the comparative analysis, the relative abundance of *Mollicutes* was over 5 and 8 log fold higher in the adult oyster gut than in adult biodeposits and larvae homogenate, respectively (ANCOM-BC, *P* value < 0.05). While a significantly higher abundance of *Mollicutes* was observed in the biodeposits than the larvae homogenate, the log fold change (1.7) was much lower than what was observed between the gut and larva samples. Interestingly, another bacterial class, the *Spirochaetia*, was also significantly enriched in adult oyster gut compared to the biodeposits (log fold change up to 2.9). In contrast, the *Gammaproteobacteria* were significantly more abundant in the larvae homogenate than the adult gut (log fold change between 2.7 and 3.0) and the biodeposits (log fold change of 3.9).

10.1128/mSphere.00227-21.5 FIG S5 Heatmap showing the differential relative abundance of the major taxa among different eastern oyster microbiome studies. Four distinct eastern oyster microbiome data sets were included in the comparison, including the adult gut samples from this study and a previous study in Long Island Sound (39), larvae homogenate samples (25), and biodeposit samples (40). The column labels (on the bottom of each heatmap) represent reference studies, and the row labels represent (on the left of each heatmap) target studies of the comparison. Positive log fold changes in the heatmap represent a higher relative abundance of a taxon in the target than the reference study and vice versa. Pairwise statistical significance was assessed with ANCOM-BC: *, *P* value < 0.05; **, *P* value < 0.01; ***, *P* value < 0.001. Download FIG S5, TIF file, 1.5 MB. Copyright © 2021 Pimentel et al. 2021 Pimentel et al. https://creativecommons.org/licenses/by/4.0/ This content is distributed under the terms of the Creative Commons Attribution 4.0 International license.

To further elucidate the taxonomic distribution of the *Mollicutes* ASVs recovered from oyster tissues and the surrounding water, a phylogenetic analysis was performed by placing the *Mollicutes* ASVs from this study and a previous study of the adult eastern oyster gut microbiome (39) into a reference tree of 16S rRNA gene sequences from the SILVA database, derived from environmental clones and organisms in pure culture (Materials and Methods). Branches in the tree were collapsed with labels indicating the dominant environmental source of the SILVA references. A bubble plot was included on the right of the phylogeny to indicate the number of unique *Mollicutes* ASVs identified from different tissue or water samples (Fig. 4).

The *Mollicutes* ASVs identified from oyster tissues were largely divided into four major clades of the phylogenetic tree (Fig. 4 and Data Set S2E): (i) Sydney rock oyster and water (clade I), including ASVs mapped to a clade of uncultured references taxonomically assigned to the family *Mycoplasmataceae*; (ii) crustaceans and soil (clade II), including ASVs mapped to a clade containing reference sequences from “*Candidatus* Bacilloplasma,” “*Candidatus* Lumbricincol,” and unidentified members of the family *Mycoplasmataceae*; (iii) water, sediment, and feces (clade III), including ASVs mapped to a clade of references from *Izemoplasmatales*, *Acholeplasmatales*, and RF39; and (iv) marine invertebrates (clade IV), including ASVs mapped to a clade of *Mycoplasma*. The highest number of unique *Mollicutes* ASVs across all eastern oyster tissues was observed in the clade IV (marine invertebrates), as shown both in this study and by Pierce and Ward (39). This clade also included the metagenome-assembled 16S rRNA genes, *Mollicutes-1* and *Mollicutes-2*, and their corresponding ASVs (Data Set S2E). In contrast, the *Mollicutes* ASVs identified from surrounding water samples were mainly placed into clade III (water, sediment, and feces) of the phylogeny. Interestingly, the gut, compared to any other tissues, included the highest number of unique *Mollicutes* ASVs in clade IV but the lowest number of unique ASVs in clade III. Overall, a high level of phylogenetic diversity was revealed within the eastern oyster-associated *Mollicutes*.

## DISCUSSION

The microbiome of marine invertebrates has potential significance in mediating the biological and ecological functions of host organisms (41, 42). A comprehensive understanding of host-microbiome interactions relies on the taxonomic and functional characterization of the microbiome across different tissue types and individuals. Despite potential encounters with the highly diverse microbial communities in the surrounding water column through their filter-feeding lifestyle, eastern oysters have been shown to generally contain low microbial diversity compared to the surrounding water column (18). While the taxonomic structure of the oyster microbiome has been previously studied, little is known about its functional potential. This study, to the best of our knowledge, represents the first metagenome-derived functional characterization of the *Mollicutes* bacteria that is prevalent in the oyster microbiome.

The metagenomic sequencing was complemented with amplicon-based 16S rRNA gene profiling across six distinct tissue types. Analyses of the alpha diversity revealed that the oysters carry a highly diverse microbiome both across different tissue types and when the same tissue type was examined among individual oysters (Fig. 1 and also Fig. S1 in the supplemental material). Significant distinctions in the microbiome composition were also observed in different oyster tissues, with the gut carrying a distinct microbiome from all other tissues examined and the oyster mantle, gill, and inner shell samples being significantly different from one another (Data Set S1C). Interestingly, the gill and mantle demonstrated significant differences in the microbiome composition despite their close proximity within the oyster, indicating tissue-specific differences in community structure and potential mechanistic differences in microbiome associations with the mantle and the gill of oysters.

Taxonomic assignments of amplicon sequences across the six profiled tissue types revealed five major taxa at the class level, including *Mollicutes*, *Chlamydiae*, *Spirochaetia*, *Fusobacteriia*, and *Gammaproteobacteria*. All of these taxa have been observed in other eastern oyster microbiome studies in various relative abundances (15, 39, 43). The presence of these major taxa was further confirmed with metagenomic sequencing of the oyster gut microbiome, where at least one full-length 16S rRNA gene was reconstructed for each taxon. Tissue-specific enrichment (e.g., *Mollicutes* and *Chlamydiae* in the gut and *Spirochaetia in the* mantle and gill) was observed for a number of the major taxa (Fig. 2). Interestingly, the *Mollicutes* was significantly enriched in the adult oyster gut, with an 8-fold-higher relative abundance compared to the larvae homogenate (25) and a 5-fold-higher relative abundance compared to the biodeposits (40) (Fig. S5).

The variability and abundance of the oyster microbiome can be affected by a number of biological and environmental factors, such as host genetics, health status, and diet (10, 19, 22, 23). While we recognize that this study has ultimately surveyed the microbiome of oysters from one site at one point in time, the observed high individual-level and tissue-specific microbiome variability has prompted us to examine the influence of a potential factor, the infection by a parasite, *P. marinus*, on the oyster microbiome, within that site. The oysters infected by *P. marinus* appear to carry a significantly lower microbiome alpha diversity in the inner shell than the uninfected oysters. This is interesting as the inner shell is a potential site of biofilm formation by some probiotic bacteria in oysters (11). In contrast, the comparison of other tissue types revealed no significant shifts in alpha diversity between the *P. marinus*-infected and uninfected samples. This is in line with other studies that have examined the eastern oyster microbiome during *P. marinus* infection (20, 44). At this point, it is unknown why the *P. marinus* infection would impact only microbiome alpha diversity in the inner shell. One possibility is that the inner shell microbiome is particularly impacted by changes in immune responses or metabolic state induced by *P. marinus* infection. Although *P. marinus* can be distributed through tissues in systemic infections, initial sites of infection were proposed to include the pseudofeces discharge area (45), so it is not unreasonable to speculate that mucosal immune responses of the mantle to *P. marinus* infection may have a secondary effect on some exposed bacteria uniquely present in the inner shell.

The mapping of ASVs onto full-length 16S rRNA genes obtained from metagenomic assembly also provided a unique opportunity for examining the abundance of specific bacteria among *P. marinus*-infected and uninfected samples (Fig. S2). Interestingly, the *Mollicutes* appeared to show various responses to the infection across different oyster tissues. Specifically, the *Mollicutes-1* and *Mollicutes-2* showed a 4- to 5-log-fold-higher relative abundance in the gut and the pallial fluid, respectively, of the uninfected than the *P. marinus*-infected oysters. Meanwhile, the *Mollicutes-1* had a slightly lower (1.2 log fold change) relative abundance in the inner shell of uninfected compared to infected oysters. This indicates potential complexity underlying the mechanisms of tissue-specific associations by the *Mollicutes* with the oyster host, and it is in contrast to the *Chlamydiae*, which demonstrated a statistically significant increase in the *P. marinus*-infected gill, mantle, and inner shell samples. The higher relative abundance of *Mollicutes-1* in uninfected gut samples is also in line with a prior study of the Sydney rock oyster (a Pacific species) where sequences related to *Mycoplasma* were present in the digestive gland of uninfected oysters but absent in oysters infected with the protozoan parasite Marteilia sydneyi (23).

Additional insights into the oyster gut microbiome have been achieved with the reconstruction of MAGs. Overall, two MAGs of high completeness and low contamination have been identified for the *Mollicutes* and *Chlamydiae*, and one partial MAG has been identified from the *Spirochetes*. Specifically, phylogenomic assignments and the calculation of pairwise ANI and AAI values with reference genomes suggest the *Mollicutes* and *Chlamydiae* MAGs are distinct from isolates previously described in other organisms and environmental samples (Fig. 3A and Data Set S2B and C). To further elucidate the functional potentials encoded in the oyster *Mollicutes* MAG, a genome-scale metabolic network was reconstructed. The metabolic reconstruction has revealed a heavy reliance of the oyster-associated *Mollicutes* on host-derived nutrients, with several unique metabolic pathways identified in the *Mollicutes* MAG compared to other neighboring strains in the phylogeny. One is a chitin utilization pathway, which supports the degradation of chitin for carbon and energy metabolism; another is a complete ADI pathway that could fuel ATP production through the utilization of arginine, an abundant amino acid in the eastern oyster (46). Interestingly, despite the presence of a GlcNAc utilization pathway in M. marinum and *M. todarodis* and the presence of an arginine deiminase gene in *M. mobile*, the complete ADI and chitin degradation pathways were present only in the oyster *Mollicutes* MAG (Fig. 3B). Arginine has been previously implicated in the infection dynamics of oysters. For example, *P. marinus* is speculated to sequester arginine to avoid host immune responses mediated by the production of nitric oxide, for which arginine is a precursor (47). The potential competition of *P. marinus* and *Mollicutes* in the utilization of host-derived arginine may provide some insights into the observed decrease of *Mollicutes* in the *P. marinus*-infected gut samples. However, the variable differential abundance of *Mollicutes* in other tissue types (e.g., pallial fluid or inner shell) indicates a potentially complex relationship between *P. marinus*, *Mollicutes*, and the oyster host that requires further investigations in future studies.

The prevalence of *Mollicutes* in the adult oyster gut is commonly observed among other 16S rRNA gene profiling studies (16, 48). Electron-dense bodies that resemble strains of the genus *Mycoplasma* are also demonstrated by transmission electron microscopy in eastern oyster gut goblet cells (49). Phylogenetic placement of *Mollicutes* ASVs among reference 16S rRNA genes of *Mollicutes* from laboratory isolates and environmental samples further elucidated a high level of phylogenetic diversity of this oyster-associated taxon (Fig. 4). The oyster-associated *Mollicutes* have been primarily identified from four distinct clades. Clade IV contains the highest number of unique ASVs across different oyster tissues and is taxonomically assigned to the genus *Mycoplasma*. Clades I and II similarly contain oyster-associated *Mollicutes* from all tissue types, but they formed distant branches from clade IV in the phylogeny and are taxonomically assigned to “*Candidatus* Bacilloplasma,” “*Candidatus* Lumbricincol,” and uncultured members of the family *Mycoplasmataceae*. While clades I, II, and IV all include references from invertebrate hosts, clade III is largely represented by free-living references from water and sediment samples and is taxonomically assigned to the *Izemoplasmatales*, *Acholeplasmatales*, and RF39 (Data Set S2E). Distinctions between clade III and other oyster-associated *Mollicutes* clades are also reflected in the higher presence of clade III *Mollicutes* ASVs in the surrounding seawater. In contrast, the three other clades had little or no presence of *Mollicutes* ASVs from the surrounding seawater. Overall, the integrated study of the phylogenetic diversity and functional potential of the eastern oyster-associated *Mollicutes* will set the stage for future research on bacterial transmission dynamics, host range, and relative impacts on host health. Further study of the mechanisms of the *Mollicutes* acquisition, persistence, and physiology will begin to shed light on the nature of the relationship between the oyster host and *Mollicutes*.

## MATERIALS AND METHODS

### Sample collection and dissection.

Twenty-nine live oysters, 1 to 3 years in age, were sampled from an aquaculture farm along with 1 liter of surface water in Ninigret Pond, Rhode Island (USA), on 25 August 2017. In this farm, eastern oysters are farmed using a rack and bag culture system, with racks suspended approximately 1.5 m from the bottom of the pond. Environmental conditions at the site were measured as part of another study (15). Oysters were transported to the laboratory in coolers on ice. Upon arrival at the laboratory, the outer shells of individual oysters were scrubbed to remove visible sediments, and the length, width, and mass of each oyster were recorded (see Data Set S1A in the supplemental material). The oysters were sprayed with 70% ethanol followed by the immediate dissection to obtain the pallial fluid, hemolymph, mantle, gill, gut, and inner shell samples. Hemolymph and pallial fluid were collected for 9 of the 29 oysters (fluid volumes recorded in Data Set S1A). The pallial fluid was collected prior to shucking by creating a small notch in the shell and using a sterile syringe and needle through the notch. Following shucking, hemolymph was obtained with a sterile syringe from the adductor muscle sinus (50). Pallial fluid and hemolymph samples were centrifuged at 16,100 × *g* for 8 min, the supernatant was removed, and the pellet was resuspended in 500 μl of RNAlater (Invitrogen, Carlsbad, CA). For all of the oysters, samples of the gut (consisting of the digestive gland, intestine, and stomach tissue), gill, mantle, and inner shell (taken from the inner surface of the top and bottom shells using sterile cotton swabs) were stored individually in 1 ml of RNAlater (Invitrogen, Carlsbad, CA). A mixed sample of gill, mantle, and rectum tissues was also preserved for each oyster in 100% ethanol for protozoan pathogen quantification (described below). The water sample was prefiltered through a 153-μm mesh filter, followed by filtration through a 5-μm-pore-size (diameter of 47 mm) filter (Sterlitech, Kent, WA) and then a 0.2-μm-pore-size Sterivex filter (Millipore, Burlington, MA). All samples were stored in a −80°C freezer before further processing. Detailed tracking of all samples and their application in the 16S rRNA community profiling and metagenomic sequencing is provided in Data Set S1A.

### Protozoan pathogen quantification.

For each of the 29 oysters, DNA was extracted from the 100% ethanol-preserved gill, mantle, and rectum samples (described above) using a previously described protocol with modifications in order to assess the abundance of the eastern oyster protozoan pathogens *Perkinsus marinus* (causative agent of Dermo disease), *Haplosporidium nelsoni* (causative agent of MSX disease), and *Haplosporidium costale* (causative agent of SSO disease) (51). First, a total of 5 mg sample was pooled from all three tissue types and was sterilely placed into a 1.5-ml microcentrifuge tube with 160 μl of preheated urea buffer and incubated at 60°C in a dry bath for 1 h. Samples were vortexed intermittently throughout the incubation. After incubation, the tubes were vortexed again and placed into a 95°C dry bath for 15 min. Samples underwent centrifugation for 5 min at 15,000 × *g*, and 100 μl of the supernatant was removed and mixed with 1 μl of 100× Tris-EDTA (TE) buffer, 50 μl ammonium acetate, and 400 μl of 95% ethanol. After mixing, the samples were left at −20°C overnight to precipitate. The following day, samples underwent centrifugation for 20 min at 15,000 × *g* to pellet the precipitate and were washed three times with 70% ice-cold ethanol to remove any remaining impurities. DNA was eluted in 100 μl of buffer (10 mM Tris-HCl, 0.1 mM EDTA, pH 8.0). Quantity and quality of the DNA were assessed with the NanoDrop 2000c (Thermo Fisher Scientific, Waltham, MA), and the samples were diluted to 100 ng in 3 μl to ensure consistency between quantitative PCRs (qPCRs).

Subsequently, the DNA was used as a template in a multiplex, real-time PCR testing for *P. marinus*, *H. nelsoni*, and *H. costale*. *P. marinus* primers and dually labeled probes from reference 31 were used in conjunction with MSX/SSO primers, MSX probe, and SSO probe (primer and probe sequences in Table 1). Each reaction with a 20-μl reaction mixture was carried out using 300 nM *P. marinus* primers, 450 nM *Haplosporidium* primers, and 75 nM specific probe for each pathogen with 3.55 μl water, 10 μl iQ Multiplex Powermix (Bio-Rad, Hercules, CA), and 3 μl (100 ng) template DNA. The thermal cycler protocol was as follows: 95°C for 60 s, followed by 40 cycles of 95°C for 15 s, then 60°C for 30 s. The qPCR copy number was used to determine the Mackin rating of each oyster following protocols adapted from reference 31. Copy numbers of the small-subunit (SSU) rRNA gene for *H. costale* and *H. nelsoni* were converted to a semiquantitative scale (0 for no infection, 1 for initial infection, 2 for moderate infection, and 3 for severe infection) adapted from reference 52.

**TABLE 1:** Primers and probes used in this study^*a*^

Target	Type	Direction	Sequence
16S rRNA gene (V4 region)	Primer	515F	5′ **TCGTCGGCAGCGTCAGATGTGTATAAGAGACAG**GTGYCAGCMGCCGCGGTAA
		806R	5′ **GTCTCGTGGGCTCGGAGATGTGTATAAGAGACAG**GGACTACNVGGGTWTCTAAT
*Perkinsus marinus* ITS	Primer	F	5′ CGCCTGTGAGTATCTCTCGA
		R	5′ GTTGAAGAGAAGAATCGCGTGAT
MSX/SSO	Primer	F	5′ ACAGGTCAGTGATGCCCTTAG
		R	5′ TSGRGATTACCYSGCCTTC
MSX	Probe		5′ 5HEX/TTGCACGCAACGAGTTCAACCTTGCCTG/3BHQ1
SSO	Probe		5′ 5Cy5/AATGACCCAGTCAGCGGGCCGA/3BHQ1
*P. marinus*	Probe		5′ 56-FAM/CGCAAACTCGACTGTGTTGTGGTG/3BHQ1

a The boldface sequences in 515F and 806R represent an overhang used for library preparation whereas the nonboldface sequence indicates the locus-specific part of the primers. The direction of each primer is shown, with F indicating the forward primer and R indicating the reverse primer. HEX, hexachloro fluorescein; Cy5, cyanine 5; 6-FAM, 6-carboxyfluorescein; BHQ, black hole quencher.

### DNA extraction.

DNA extractions were performed with the Qiagen Allprep PowerFecal DNA/RNA kit (Qiagen, Hilden, Germany) following the manufacturer’s protocol with slight modifications, including the addition of 2 μl of proteinase K (Thermo Fisher Scientific, Waltham, MA) and 13.5 μl of 20% SDS (Thermo Fisher Scientific, Waltham, MA) to the lysis buffer. Mechanical lysis was performed via two rounds of homogenization with a TissueLyser (Qiagen, Hilden, Germany) for 2.5 min each at 30 Hz, and the samples were incubated for 5 min at room temperature in between. Samples included approximately 30 mg each of gut, gill, and mantle tissue as well as all the collected hemolymph, pallial fluid, and inner shell samples from each oyster (Data Set S1A). Additionally, one preparation of the ZymoBIOMICS Microbial Community Standard (Zymo, Irvine, CA) was extracted with the same protocol and used as a reference for quality control in the amplicon sequencing. The DNA from the 0.2- and 5-μm-pore-size filters from the water samples was extracted with the Qiagen QIAamp PowerFecal DNA kit following the manufacturer’s protocol with the same modifications as above. However, the TissueLyser was used only for a total of 1 min (30 s at 30 Hz, incubated for 5 min at room temperatures, and then 30 s at 30 Hz). A PVC pipe cutter was used to open the Sterivex filter, and a sterile scalpel and forceps were used to remove half of the filter sample for DNA extraction (53).

### 16S rRNA community profiling.

16S rRNA gene profiling was performed on the oyster tissue samples, the ZymoBIOMICS Microbial Community Standard sample, and the water samples collected at the time of sampling. Template DNA was amplified with V4 primers 515F/806R with Illumina adapter overhangs (Table 1) and 2× Phusion HF master mix (Thermo Fisher Scientific, Waltham, MA) (54, 55). The PCR was configured in a touchdown protocol and contained the following program: 3-min initial denaturation at 94°C, followed by 10 cycles of a 45-s denaturation at 94°C, 60-s annealing at 60°C with every cycle decreasing 1°C, and 30 s of elongation at 72°C, followed by 25 cycles of a 45-s denaturation at 94°C, 60-s annealing at 50°C, and 30 s of elongation at 72°C; 10 min of final extension at 72°C; and an indefinite hold at 4°C. Sequencing libraries were prepared at the Rhode Island Genomics and Sequencing Center (RIGSC) using a standardized protocol as follows: 50 ng of AMPure XP-cleaned PCR product was used as a template in a second PCR (5 cycles) with 2× Phusion HF master mix in order to attach full indices and adapters with the Illumina Nextera index kit (Illumina, San Diego, CA). The resultant PCR products were cleaned with AMPure XP, profiled with an Agilent Bioanalyzer DNA1000 chip (Agilent Technologies, Santa Clara, CA), quantified using a Qubit fluorometer (Invitrogen, Carlsbad, CA), and normalized for pooling. Then, the pooled library was quantified with qPCR with a Roche LightCycler 480 using the KAPA Biosystems Illumina kit (KAPA Biosystems, Wilmington, MA). The final pooled library underwent two rounds of 2 × 250-bp sequencing on the same Illumina MiSeq instrument (Illumina, San Diego, CA).

### Microbiome enrichment of pooled gut samples.

In order to enhance the metagenomic binning of coassembled contigs, a microbial cell enrichment procedure was performed on the gut samples through the adaptation of a procedure previously applied to marine sponges (56). The gut samples of 10 oysters were pooled and homogenized with 10 ml of ice-cold, sterile artificial seawater (28 ppt) in an autoclaved mortar and pestle and vortexed for 10 min. The homogenized sample was centrifuged at 100 × *g* at 4°C for 30 min. The supernatant was extracted and centrifuged again at 10,967 × *g* at 4°C for 30 min. After removing the supernatant, the resultant pellet was washed three times with ice-cold, sterile artificial seawater. The pellet from the final round of centrifugation was diluted with ice-cold, sterile artificial seawater and pushed through a 3-μm-pore-size (diameter of 47 mm) filter (Millipore, Burlington, MA) with a syringe. The flowthrough was subsequently pushed through a 0.2-μm-pore-size (diameter of 22 mm) filter (Sterlitech, Kent, WA). DNA extraction was then performed on the 0.2-μm filter using the Qiagen QIAamp PowerFecal DNA kit following manufacturer protocols. Mechanical cell lysis was performed via two rounds of homogenization with a TissueLyser (Qiagen, Hilden, Germany) for 1 min each at 30 Hz, and the sample was incubated for 5 min at room temperature in between.

### Shotgun metagenomic sequencing.

Two rounds of metagenomic sequencing were performed (Data Set S1A). In the first round, 12 gut samples were individually barcoded and prepared for shotgun metagenomics using a read length of 2 × 150 bp across three lanes on an Illumina HiSeq 4000. In the second round, one microbiome-enriched oyster gut sample was prepared using the microbiome enrichment protocol described above and barcoded for shotgun metagenomics using a read length of 2 × 150 bp on an Illumina NextSeq 500 (mid-output). Preparation of metagenomic libraries was performed at the RIGSC using standardized protocols including DNA fragmentation with an S220 ultrasonicator (Covaris, Woburn, MA), quantification with a Qubit fluorometer (Invitrogen, Carlsbad, CA), and library preparation (including end repair, A-tailing, adapter ligation, and size selection) on the Wafergen Apollo 324 with PrepX reagents and consumables (TaKaRa Bio, Kusatsu, Japan), followed by quality control using an Agilent Bioanalyzer high-sensitivity chip (Agilent Technologies, Santa Clara, CA) and quantification of the final library with qPCR with a Roche LightCycler 480 using the KAPA Biosystems Illumina kit (KAPA Biosystems, Wilmington, MA). Sequencing of the metagenomic libraries was performed at Duke University’s Sequencing and Genomic Technologies Core.

### Amplicon sequence analysis.

Demultiplexed read pairs from the two MiSeq runs were analyzed independently using the Quantitative Insights Into Microbial Ecology 2 (QIIME2) software version 2018.4 (57). First, the raw reads were imported using the *qiime tools import* function, and the read quality was inspected for the selection of trimming parameters using the *qiime demux summarize* function. Using the DADA2 plugin, samples were subjected to quality filtering, merging, denoising, and chimera detection with the *qiime dada2 denoise-paired* function trimming the forward and reverse reads at 20 bp at the 5′ end, the forward reads at 230 bp at the 3′ end, and the reverse reads at 200 bp at the 3′ end (58). This step led to the establishment of a count table that maps the occurrence of ASVs in each sample. The ASV count tables from the two different MiSeq runs were merged using the *qiime feature-table merge* function in QIIME2 using the sum overlap method specified with the argument *–p-overlap-method sum*. All subsequent steps were performed on the combined MiSeq run data. Following quality control and pooling of the two sequencing runs, samples with less than 10,000 total read pairs were dropped from further analysis due to low sequencing depth (Data Set S1A).

Taxonomic assignment was performed with QIIME2’s sklearn classifier by mapping to the SILVA database release 132 (59). Mitochondrial and chloroplast sequences were removed from the ASV count table based on the SILVA taxonomic assignments. The resulting ASV count table and taxonomy data were exported and analyzed in R version 4.0.2. The taxonomic assignments were used to validate the identification of all 8 bacterial members (3 Gram negative and 5 Gram positive) in the ZymoBIOMICS Microbial Community Standard sample, suggesting adequate cell lysis during DNA extraction. The Shannon and Simpson indices were computed based on the ASV count table with the *diversity* function in the vegan package version 2.5-6 (60). Statistical significance in the ASV counts, Shannon index, and Simpson index comparisons was evaluated using the *pairwise.wilcox.test* function in the core stats library in R with a Holm *P* value adjustment. Unless specified, the comparisons of alpha diversity, beta diversity, and taxonomic relative abundance were performed on all the oyster samples that underwent amplicon sequencing.

To account for the compositional nature of the ASV count table (61, 62), a phylogenetic isometric log-ratio transformation was performed with the PhILR package version 1.16.0 (63). The ASV phylogeny used in the PhILR analysis was constructed following multiple sequence alignment with MAFFT (64), alignment masking with the *qiime alignment mask* function, phylogenetic reconstruction with FastTree (65), and midpoint rooting in QIIME2. The PhILR-transformed ASV counts were used to compute between-sample distances using the Euclidean distance measure. Pairwise PERMANOVAs were conducted on the between-sample distances with the R package pairwiseAdonis version 0.4 to assess the statistical significance in comparisons of microbiome composition (66). Differential abundance of specific taxa was evaluated among the different tissue types and between *P. marinus*-infected and uninfected samples using the *ancombc* function with default parameters in the analysis of compositions of microbiomes with bias correction (ANCOM-BC) package, version 1.0.2, and the heatmap visualizations were constructed using the *geom_tile* function in ggplot2 to demonstrate the log fold changes and statistical significance (32). For the comparative analysis to prior studies, raw reads from three published eastern oyster microbiome data sets were retrieved from the NCBI Sequence Read Archive (SRA) linked to the BioProject accession numbers PRJNA518081 (25), PRJNA504404 (40), and PRJNA386685 (39) and subjected to the analytical protocols as specified above.

### Metagenomic assembly and binning.

The data from both rounds of metagenomic sequencing underwent trimming and adapter removal with Trimmomatic version 0.38 (leading and trailing bases below a quality score of 3 were removed, a 4-bp sliding window was used with an average quality score of 15, and a minimum read length of 130 bp was required), and the last 10 bp of all reads were removed with Cutadapt version 1.9.1 (67, 68). Due to differences in the chemistry associated with the Illumina NextSeq and HiSeq platforms, the reads from the two rounds of metagenomic sequencing underwent slightly different quality control measures. The reads from the sample that was sequenced on the Illumina NextSeq 500 underwent additional steps in Trimmomatic and Cutadapt in order to remove poly(G). In Trimmomatic, a sequence composed of 50 guanine residues was added to the adapter sequences, and a sequence of 10 guanine residues was used as an adapter sequence in Cutadapt (allowing for an error rate of 20%) to enable the removal of poly(G) sequences at the 3′ end of reads.

The quality-filtered reads were then mapped with BBMap version 37.36 to a combined reference database containing the *C. virginica* genome (RefSeq assembly accession GCF_002022765.2), the *P. marinus* genome (RefSeq assembly accession GCF_000006405.1), all complete bacterial genomes downloaded on 18 April 2018, and a collection of manually curated draft genomes of bacteria isolated from oysters (strain names and NCBI RefSeq accession numbers in Data Set S2F). Reads mapped to a bacterial genome or unmapped to any genomes in the reference database were used for the metagenomic coassembly and binning. Paired reads were considered mapped to a bacterial genome if at least one read from the pair was mapped. Metagenomic coassembly was performed using MEGAHIT version 1.1.1 with default parameters (69). Binning of the coassembled contigs was performed with default parameters in MetaBat2 version 2.12.1 (70) using read mappings performed with BBMap, with the resulting SAM files converted to BAM format and sorted with SAMtools release 1.5 (71). Completeness and contamination of the MAGs reconstructed from the oyster microbiome were assessed using CheckM version 1.0.5. The *ssu_finder* function in CheckM was used to identify 16S rRNA genes in the metagenomic coassembly (72). Candidate 16S rRNA genes greater than 1,000 bp in length were retained.

### Phylogenetic positioning of *Mollicutes* ASVs.

All ASVs taxonomically assigned to the class *Mollicutes* from this and a prior study (39), two full-length *Mollicutes* 16S rRNA genes assembled from the metagenomes collected in this study, and 11 full-length 16S rRNA genes obtained from the *Firmicutes* outgroup (Data Set S2E) were placed into a reference tree with SILVA’s ACT server, using the SINA aligner (73). The minimum identity was set at 85%, and the number of neighbors per query was set at 5. The resultant phylogeny was collapsed based on the environment where the reference SILVA sequences were derived in iTOL (74).

### Phylogenomic analyses of *Mollicutes* and *Chlamydiae* MAGs.

Phylogenomic reconstructions were performed on the *Mollicutes* MAG and the *Chlamydiae* MAG with reference genomes from the two corresponding taxa (Data Set S2B and C). To ensure consistency in gene calling, all genomes were first analyzed using Prodigal version 2.6.3 (75) with genetic code 4 for the *Mollicutes* and standard genetic code for the *Chlamydiae*. Conserved single-copy genes (CSCGs) were identified through the analysis of bidirectional best BLAST hits as described in a previous study (76). The identified CSCGs were individually aligned with MUSCLE version 3.8.31, the alignments were concatenated, and a phylogeny was reconstructed with RAxML version 8.2.10 using the JTT substitution model and the GAMMA model of rate heterogeneity. Pairwise average nucleotide identity (ANI) values were computed with OrthANI version 1.40 (77). The average amino acid identity (AAI) was computed by the mean protein identity values of all bidirectional best BLAST hits identified based on the following thresholds: E value less than or equal to 1e−3, sequence identity greater than or equal to 30%, and coverage of 70% or higher to both sequences in the alignment.

### Metabolic reconstruction of the *Mollicutes* MAG.

A metabolic reconstruction of the *Mollicutes* MAG was developed based on an initial mapping of the proteome to the KEGG database through the KAAS server (78), and the annotation of transporters was based on homology searches to the Transporter Classification Database (79). The draft reconstruction underwent manual curations through comparisons to three *Mycoplasma* isolates closely related to the *Mollicutes* MAG (i.e., *M. mobile*, *M. todarodis*, and M. marinum) and using reference annotations from an existing metabolic model of Mycoplasma pneumoniae (80). The metabolic reconstruction was incorporated into a genome-scale model using PSAMM (81). Metabolic pathway gaps were identified using the PSAMM *gapcheck* function, which in turn were used to guide the manual curation of gene functions from the MAG and other contigs identified from the metagenomic coassembly. For example, the pyruvate dehydrogenase complex was initially not identified in the *Mollicutes* MAG, but its subunits were found on other contigs in the coassembly with top BLAST hits (in the NCBI nonredundant protein database) to members of the *Mycoplasma* genus. In this case, the pyruvate dehydrogenase reaction was included in the metabolic model to highlight the potential presence of this function. Some gap reactions, such as the transport of acetate, were included in the metabolic network to represent the potential export of acetate as a metabolic product.

### Data availability.

Raw sequencing data are available from the NCBI Sequence Read Archive under the BioProject accession PRJNA658576.

## References

[1] Peterson CH, Grabowski JH, Powers SP. 2003. Estimated enhancement of fish production resulting from restoring oyster reef habitat: quantitative valuation. Mar Ecol Prog Ser 264:249–264. doi:10.3354/meps264249.

[2] Piazza BP, Banks PD, La Peyre MK. 2005. The potential for created oyster shell reefs as a sustainable shoreline protection strategy in Louisiana. Restor Ecol 13:499–506. doi:10.1111/j.1526-100X.2005.00062.x.

[3] Caffrey JM, Hollibaugh JT, Mortazavi B. 2016. Living oysters and their shells as sites of nitrification and denitrification. Mar Pollut Bull 112:86–90. doi:10.1016/j.marpolbul.2016.08.038. 27567196

[4] Huanxin W, Lejun Z, Presley BJ. 2000. Bioaccumulation of heavy metals in oyster (Crassostrea virginica) tissue and shell. Environ Geol 39:1216–1226. doi:10.1007/s002540000110.

[5] Humphries AT, Ayvazian SG, Carey JC, Hancock BT, Grabbert S, Cobb D, Strobel CJ, Fulweiler RW. 2016. Directly measured denitrification reveals oyster aquaculture and restored oyster reefs remove nitrogen at comparable high rates. Front Mar Sci 3:74. doi:10.3389/fmars.2016.00074.

[6] FAO. 2020. The state of world fisheries and aquaculture 2020: sustainability in action. Food and Agriculture Organization of the United Nations, Rome, Italy. doi:10.4060/ca9229en.

[7] Pierce ML, Ward JE. 2018. Microbial ecology of the Bivalvia, with an emphasis on the family Ostreidae. J Shellfish Res 37:793–806. doi:10.2983/035.037.0410.

[8] Blackstone GM, Nordstrom JL, Vickery MCL, Bowen MD, Meyer RF, DePaola A. 2003. Detection of pathogenic Vibrio parahaemolyticus in oyster enrichments by real time PCR. J Microbiol Methods 53:149–155. doi:10.1016/S0167-7012(03)00020-4. 12654486

[9] Wang R, Zhong Y, Gu X, Yuan J, Saeed AF, Wang S. 2015. The pathogenesis, detection, and prevention of Vibrio parahaemolyticus. Front Microbiol 6:144. doi:10.3389/fmicb.2015.00144. 25798132 PMC4350439

[10] Wegner KM, Piel D, Bruto M, John U, Mao Z, Alunno-Bruscia M, Petton B, Le Roux F. 2019. Molecular targets for coevolutionary interactions between Pacific oyster larvae and their sympatric vibrios. Front Microbiol 10:2067. doi:10.3389/fmicb.2019.02067. 31555250 PMC6742746

[11] Karim M, Zhao W, Rowley D, Nelson D, Gomez-Chiarri M. 2013. Probiotic strains for shellfish aquaculture: protection of Eastern oyster, Crassostrea virginica, larvae and juveniles against bacterial challenge. J Shellfish Res 32:401–408. doi:10.2983/035.032.0220.

[12] Lim HJ, Kapareiko D, Schott EJ, Hanif A, Wikfors GH. 2011. Isolation and evaluation of new probiotic bacteria for use in shellfish hatcheries: I. Isolation and screening for bioactivity. J Shellfish Res 30:609–615. doi:10.2983/035.030.0303.

[13] Smolowitz R. 2013. A review of current state of knowledge concerning Perkinsus marinus effects on Crassostrea virginica (Gmelin) (the eastern oyster). Vet Pathol 50:404–411. doi:10.1177/0300985813480806. 23462867

[14] Fernández Robledo JA, Yadavalli R, Allam B, Pales Espinosa E, Gerdol M, Greco S, Stevick RJ, Gómez-Chiarri M, Zhang Y, Heil CA, Tracy AN, Bishop-Bailey D, Metzger MJ. 2019. From the raw bar to the bench: bivalves as models for human health. Dev Comp Immunol 92:260–282. doi:10.1016/j.dci.2018.11.020. 30503358 PMC6511260

[15] Stevick RJ, Post AF, Gómez-Chiarri M. 2021. Functional plasticity in oyster gut microbiomes along a eutrophication gradient in an urbanized estuary. Anim Microbiome 3:5. doi:10.1186/s42523-020-00066-0. 33499983 PMC7934548

[16] King GM, Judd C, Kuske CR, Smith C. 2012. Analysis of stomach and gut microbiomes of the eastern oyster (Crassostrea virginica) from coastal Louisiana, USA. PLoS One 7:e51475. doi:10.1371/journal.pone.0051475. 23251548 PMC3520802

[17] Fernandez-Piquer J, Bowman JP, Ross T, Tamplin ML. 2012. Molecular analysis of the bacterial communities in the live Pacific oyster (Crassostrea gigas) and the influence of postharvest temperature on its structure. J Appl Microbiol 112:1134–1143. doi:10.1111/j.1365-2672.2012.05287.x. 22429335

[18] Lokmer A, Kuenzel S, Baines JF, Wegner KM. 2016. The role of tissue-specific microbiota in initial establishment success of Pacific oysters. Environ Microbiol 18:970–987. doi:10.1111/1462-2920.13163. 26695476

[19] Lokmer A, Mathias Wegner K. 2015. Hemolymph microbiome of Pacific oysters in response to temperature, temperature stress and infection. ISME J 9:670–682. doi:10.1038/ismej.2014.160. 25180968 PMC4331581

[20] Pierce ML, Ward JE, Holohan BA, Zhao X, Hicks RE. 2016. The influence of site and season on the gut and pallial fluid microbial communities of the eastern oyster, Crassostrea virginica (Bivalvia, Ostreidae): community-level physiological profiling and genetic structure. Hydrobiologia 765:97–113. doi:10.1007/s10750-015-2405-z.

[21] Britt A, Bernini M, McSweeney B, Dalapati S, Duchin S, Cavanna K, Santos N, Donovan G, O’Byrne K, Noyes S, Romero M, Poonacha KNT, Scully T. 2020. The effects of atrazine on the microbiome of the eastern oyster: Crassostrea virginica. Sci Rep 10:11088. doi:10.1038/s41598-020-67851-4. 32632188 PMC7338443

[22] Simons AL, Churches N, Nuzhdin S. 2018. High turnover of faecal microbiome from algal feedstock experimental manipulations in the Pacific oyster (Crassostrea gigas). Microb Biotechnol 11:848–858. doi:10.1111/1751-7915.13277. 29749083 PMC6116748

[23] Green TJ, Barnes AC. 2010. Bacterial diversity of the digestive gland of Sydney rock oysters, Saccostrea glomerata infected with the paramyxean parasite, Marteilia sydneyi. J Appl Microbiol 109:613–622. doi:10.1111/j.1365-2672.2010.04687.x. 20202017

[24] King WL, Jenkins C, Seymour JR, Labbate M. 2019. Oyster disease in a changing environment: decrypting the link between pathogen, microbiome and environment. Mar Environ Res 143:124–140. doi:10.1016/j.marenvres.2018.11.007. 30482397

[25] Stevick RJ, Sohn S, Modak TH, Nelson DR, Rowley DC, Tammi K, Smolowitz R, Lundgren KM, Post AF, Gómez-Chiarri M. 2019. Bacterial community dynamics in an oyster hatchery in response to probiotic treatment. Front Microbiol 10:1060. doi:10.3389/fmicb.2019.01060. 31156583 PMC6530434

[26] Pita L, Rix L, Slaby BM, Franke A, Hentschel U. 2018. The sponge holobiont in a changing ocean: from microbes to ecosystems. Microbiome 6:46. doi:10.1186/s40168-018-0428-1. 29523192 PMC5845141

[27] Dubilier N, Bergin C, Lott C. 2008. Symbiotic diversity in marine animals: the art of harnessing chemosynthesis. Nat Rev Microbiol 6:725–740. doi:10.1038/nrmicro1992. 18794911

[28] Lloyd-Price J, Mahurkar A, Rahnavard G, Crabtree J, Orvis J, Hall AB, Brady A, Creasy HH, McCracken C, Giglio MG, McDonald D, Franzosa EA, Knight R, White O, Huttenhower C. 2017. Strains, functions and dynamics in the expanded Human Microbiome Project. Nature 550:61–66. doi:10.1038/nature23889. 28953883 PMC5831082

[29] Griffin TW, Baer JG, Ward JE. 2021. Direct comparison of fecal and gut microbiota in the blue mussel (Mytilus edulis) discourages fecal sampling as a proxy for resident gut community. Microb Ecol 81:180–192. doi:10.1007/s00248-020-01553-2. 32638043

[30] Mackin JG. 1962. Oyster disease caused by Dermocystidium Marinum and other microorganisms in Louisiana. Publ Inst Mar Sci Univ Tex 7:132–229.

[31] De Faveri J, Smolowitz RM, Roberts SB. 2009. Development and validation of a real-time quantitative PCR assay for the detection and quantification of Perkinsus marinus in the eastern oyster, Crassostrea virginica. J Shellfish Res 28:459–464. doi:10.2983/035.028.0306.

[32] Lin H, Peddada SD. 2020. Analysis of compositions of microbiomes with bias correction. Nat Commun 11:3514. doi:10.1038/s41467-020-17041-7. 32665548 PMC7360769

[33] Ramírez AS, Vega-Orellana OM, Viver T, Poveda JB, Rosales RS, Poveda CG, Spergser J, Szostak MP, Caballero MJ, Ressel L, Bradbury JM, Mar Tavío M, Karthikeyan S, Amann R, Konstantinidis KT, Rossello-Mora R. 2019. First description of two moderately halophilic and psychrotolerant Mycoplasma species isolated from cephalopods and proposal of Mycoplasma marinum sp. nov. and Mycoplasma todarodis sp. nov. Syst Appl Microbiol 42:457–467. doi:10.1016/j.syapm.2019.04.003. 31072660

[34] Michel R, Müller KD, Zoeller L, Walochnik J, Hartmann M, Schmid EN. 2005. Free-living amoebae serve as a host for the Chlamydia-like bacterium Simkania negevensis. Acta Protozool 44:113–121.

[35] Lieberman D, Kahane S, Lieberman D, Friedman MG. 1997. Pneumonia with serological evidence of acute infection with the Chlamydia-like microorganism “Z.” Am J Respir Crit Care Med 156:578–582. doi:10.1164/ajrccm.156.2.9608081. 9279243

[36] Konstantinidis KT, Tiedje JM. 2005. Towards a genome-based taxonomy for prokaryotes. J Bacteriol 187:6258–6264. doi:10.1128/JB.187.18.6258-6264.2005. 16159757 PMC1236649

[37] Goris J, Konstantinidis KT, Klappenbach JA, Coenye T, Vandamme P, Tiedje JM. 2007. DNA-DNA hybridization values and their relationship to whole-genome sequence similarities. Int J Syst Evol Microbiol 57:81–91. doi:10.1099/ijs.0.64483-0. 17220447

[38] Noens EEE, Lolkema JS. 2017. Convergent evolution of the arginine deiminase pathway: the ArcD and ArcE arginine/ornithine exchangers. Microbiologyopen 6:e00412. doi:10.1002/mbo3.412. PMC5300872 27804281

[39] Pierce ML, Ward JE. 2019. Gut microbiomes of the eastern oyster (Crassostrea virginica) and the blue mussel (Mytilus edulis): temporal variation and the influence of marine aggregate-associated microbial communities. mSphere 4:e00730-19. doi:10.1128/mSphere.00730-19. 31826972 PMC6908423

[40] Murphy AE, Kolkmeyer R, Song B, Anderson IC, Bowen J. 2019. Bioreactivity and microbiome of biodeposits from filter-feeding bivalves. Microb Ecol 77:343–357. doi:10.1007/s00248-018-01312-4. 30612185

[41] Jones BW, Nishiguchi MK. 2004. Counterillumination in the Hawaiian bobtail squid, Euprymna scolopes Berry (Mollusca: Cephalopoda). Mar Biol 144:1151–1155. doi:10.1007/s00227-003-1285-3.

[42] van Oppen MJH, Blackall LL. 2019. Coral microbiome dynamics, functions and design in a changing world. Nat Rev Microbiol 17:557–567. doi:10.1038/s41579-019-0223-4. 31263246

[43] Arfken A, Song B, Allen SK, Carnegie RB. 2021. Comparing larval microbiomes of the eastern oyster (Crassostrea virginica) raised in different hatcheries. Aquaculture 531:735955. doi:10.1016/j.aquaculture.2020.735955.

[44] Sakowski EG. 2015. The microbiome of the eastern oyster, Crassostrea virginica, in health and disease. Ph.D. thesis. University of Delaware, Newark, DE.

[45] Allam B, Carden WE, Ward JE, Ralph G, Winnicki S, Espinosa EP. 2013. Early host-pathogen interactions in marine bivalves: evidence that the alveolate parasite Perkinsus marinus infects through the oyster mantle during rejection of pseudofeces. J Invertebr Pathol 113:26–34. doi:10.1016/j.jip.2012.12.011. 23274079

[46] Tikunov AP, Johnson CB, Lee H, Stoskopf MK, Macdonald JM. 2010. Metabolomic investigations of American oysters using 1H-NMR spectroscopy. Mar Drugs 8:2578–2596. doi:10.3390/md8102578. 21116407 PMC2992993

[47] Villamil L, Gómez-León J, Gómez-Chiarri M. 2007. Role of nitric oxide in the defenses of Crassostrea virginica to experimental infection with the protozoan parasite Perkinsus marinus. Dev Comp Immunol 31:968–977. doi:10.1016/j.dci.2007.01.006. 17368535

[48] Arfken A, Song B, Bowman JS, Piehler M. 2017. Denitrification potential of the eastern oyster microbiome using a 16S rRNA gene based metabolic inference approach. PLoS One 12:e0185071. doi:10.1371/journal.pone.0185071. 28934286 PMC5608302

[49] Harshbarger JC, Chang SC. 1977. Chlamydiae (with phages), mycoplasmas, and richettsiae in Chesapeake Bay bivalves. Science 196:666–668. doi:10.1126/science.193184. 193184

[50] Gagnaire B, Duchemin M, Auffret M, Thomas-Guyon H, Renault T. 2008. Comparison of hemocyte parameters in the pericardial cavity and the adductor muscle sinus in the Pacific oyster, Crassostrea gigas using two types of flow cytometers. Aquat Living Resour 21:39–43. doi:10.1051/alr:2008009.

[51] Aranishi F, Okimoto T. 2006. A simple and reliable method for DNA extraction from bivalve mantle. J Appl Genet 47:251–254. doi:10.1007/BF03194632. 16877805

[52] Wilbur AE, Ford SE, Gauthier JD, Gomez-Chiarri M. 2012. Quantitative PCR assay to determine prevalence and intensity of MSX (Haplosporidium nelsoni) in North Carolina and Rhode Island oysters Crassostrea virginica. Dis Aquat Org 102:107–118. doi:10.3354/dao02540. 23269385

[53] Cruaud P, Vigneron A, Fradette MS, Charette SJ, Rodriguez MJ, Dorea CC, Culley AI. 2017. Open the Sterivex^TM^ casing: an easy and effective way to improve DNA extraction yields. Limnol Oceanogr Methods 15:1015–1020. doi:10.1002/lom3.10221.

[54] Parada AE, Needham DM, Fuhrman JA. 2016. Every base matters: assessing small subunit rRNA primers for marine microbiomes with mock communities, time series and global field samples. Environ Microbiol 18:1403–1414. doi:10.1111/1462-2920.13023. 26271760

[55] Apprill A, McNally S, Parsons R, Weber L. 2015. Minor revision to V4 region SSU rRNA 806R gene primer greatly increases detection of SAR11 bacterioplankton. Aquat Microb Ecol 75:129–137. doi:10.3354/ame01753.

[56] Fieseler L, Horn M, Wagner M, Hentschel U. 2004. Discovery of the novel candidate phylum “Poribacteria” in marine sponges. Appl Environ Microbiol 70:3724–3732. doi:10.1128/AEM.70.6.3724-3732.2004. 15184179 PMC427773

[57] Bolyen E, Rideout JR, Dillon MR, Bokulich NA, Abnet CC, Al-Ghalith GA, Alexander H, Alm EJ, Arumugam M, Asnicar F, Bai Y, Bisanz JE, Bittinger K, Brejnrod A, Brislawn CJ, Brown CT, Callahan BJ, Caraballo-Rodríguez AM, Chase J, Cope EK, Da Silva R, Diener C, Dorrestein PC, Douglas GM, Durall DM, Duvallet C, Edwardson CF, Ernst M, Estaki M, Fouquier J, Gauglitz JM, Gibbons SM, Gibson DL, Gonzalez A, Gorlick K, Guo J, Hillmann B, Holmes S, Holste H, Huttenhower C, Huttley GA, Janssen S, Jarmusch AK, Jiang L, Kaehler BD, Kang KB, Keefe CR, Keim P, Kelley ST, Knights D, Koester I, Kosciolek T, Kreps J, Langille MGI, Lee J, Ley R, Liu Y-X, Loftfield E, Lozupone C, Maher M, Marotz C, Martin BD, McDonald D, McIver LJ, Melnik AV, Metcalf JL, Morgan SC, Morton JT, Naimey AT, Navas-Molina JA, Nothias LF, Orchanian SB, Pearson T, Peoples SL, Petras D, Preuss ML, Pruesse E, Rasmussen LB, Rivers A, Robeson MS, II, Rosenthal P, Segata N, Shaffer M, Shiffer A, Sinha R, Song SJ, Spear JR, Swafford AD, Thompson LR, Torres PJ, Trinh P, Tripathi A, Turnbaugh PJ, Ul-Hasan S, van der Hooft JJJ, Vargas F, Vázquez-Baeza Y, Vogtmann E, von Hippel M, Walters W, Wan Y, Wang M, Warren J, Weber KC, Williamson CHD, Willis AD, Xu ZZ, Zaneveld JR, Zhang Y, Zhu Q, Knight R, Caporaso JG. 2019. Reproducible, interactive, scalable and extensible microbiome data science using QIIME 2. Nat Biotechnol 37:852–857. doi:10.1038/s41587-019-0209-9. 31341288 PMC7015180

[58] Callahan BJ, McMurdie PJ, Rosen MJ, Han AW, Johnson AJA, Holmes SP. 2016. DADA2: high-resolution sample inference from Illumina amplicon data. Nat Methods 13:581–583. doi:10.1038/nmeth.3869. 27214047 PMC4927377

[59] Quast C, Pruesse E, Yilmaz P, Gerken J, Schweer T, Yarza P, Peplies J, Glöckner FO. 2013. The SILVA ribosomal RNA gene database project: improved data processing and web-based tools. Nucleic Acids Res 41:D590–D596. doi:10.1093/nar/gks1219. 23193283 PMC3531112

[60] Oksanen J, Blanchet FG, Friendly M, Kindt R, Legendre P, McGlinn D, Minchin PR, O’Hara RB, Simpson GL, Solymos P, Stevens MHH, Szoecs E, Wagner H. 2019. vegan: community ecology package. R package version 2.5-6.

[61] Gloor GB, Macklaim JM, Pawlowsky-Glahn V, Egozcue JJ. 2017. Microbiome datasets are compositional: and this is not optional. Front Microbiol 8:2224. doi:10.3389/fmicb.2017.02224. 29187837 PMC5695134

[62] Weiss S, Xu ZZ, Peddada S, Amir A, Bittinger K, Gonzalez A, Lozupone C, Zaneveld JR, Vázquez-Baeza Y, Birmingham A, Hyde ER, Knight R. 2017. Normalization and microbial differential abundance strategies depend upon data characteristics. Microbiome 5:27. doi:10.1186/s40168-017-0237-y. 28253908 PMC5335496

[63] Silverman JD, Washburne AD, Mukherjee S, David LA. 2017. A phylogenetic transform enhances analysis of compositional microbiota data. Elife 6:e21887. doi:10.7554/eLife.21887. 28198697 PMC5328592

[64] Katoh K, Standley DM. 2013. MAFFT multiple sequence alignment software version 7: improvements in performance and usability. Mol Biol Evol 30:772–780. doi:10.1093/molbev/mst010. 23329690 PMC3603318

[65] Price MN, Dehal PS, Arkin AP. 2010. FastTree 2 – approximately maximum-likelihood trees for large alignments. PLoS One 5:e9490. doi:10.1371/journal.pone.0009490. 20224823 PMC2835736

[66] Martinez Arbizu P. 2020 pairwiseAdonis: pairwise multilevel comparison using Adonis. R package version 0.4.

[67] Bolger AM, Lohse M, Usadel B. 2014. Trimmomatic: a flexible trimmer for Illumina sequence data. Bioinformatics 30:2114–2120. doi:10.1093/bioinformatics/btu170. 24695404 PMC4103590

[68] Martin M. 2011. Cutadapt removes adapter sequences from high-throughput sequencing reads. EMBnet J 17:10–12. doi:10.14806/ej.17.1.200.

[69] Li D, Liu C-M, Luo R, Sadakane K, Lam T-W. 2015. MEGAHIT: an ultra-fast single-node solution for large and complex metagenomics assembly via succinct de Bruijn graph. Bioinformatics 31:1674–1676. doi:10.1093/bioinformatics/btv033. 25609793

[70] Kang D, Li F, Kirton E, Thomas A, Egan RS, An H, Wang Z. 2019. MetaBAT 2: an adaptive binning algorithm for robust and efficient genome reconstruction from metagenome assemblies. PeerJ 7:e7359. doi:10.7717/peerj.7359. 31388474 PMC6662567

[71] Li H, Handsaker B, Wysoker A, Fennell T, Ruan J, Homer N, Marth G, Abecasis G, Durbin R, 1000 Genome Project Data Processing Subgroup. 2009. The Sequence Alignment/Map format and SAMtools. Bioinformatics 25:2078–2079. doi:10.1093/bioinformatics/btp352. 19505943 PMC2723002

[72] Parks DH, Imelfort M, Skennerton CT, Hugenholtz P, Tyson GW. 2015. CheckM: assessing the quality of microbial genomes recovered from isolates, single cells, and metagenomes. Genome Res 25:1043–1055. doi:10.1101/gr.186072.114. 25977477 PMC4484387

[73] Pruesse E, Peplies J, Glöckner FO. 2012. SINA: accurate high-throughput multiple sequence alignment of ribosomal RNA genes. Bioinformatics 28:1823–1829. doi:10.1093/bioinformatics/bts252. 22556368 PMC3389763

[74] Letunic I, Bork P. 2007. Interactive Tree Of Life (iTOL): an online tool for phylogenetic tree display and annotation. Bioinformatics 23:127–128. doi:10.1093/bioinformatics/btl529. 17050570

[75] Hyatt D, Chen G-L, Locascio PF, Land ML, Larimer FW, Hauser LJ. 2010. Prodigal: prokaryotic gene recognition and translation initiation site identification. BMC Bioinformatics 11:119. doi:10.1186/1471-2105-11-119. 20211023 PMC2848648

[76] Zhang Y, Sievert SM. 2014. Pan-genome analyses identify lineage- and niche-specific markers of evolution and adaptation in Epsilonproteobacteria. Front Microbiol 5:110. doi:10.3389/fmicb.2014.00110. 24678308 PMC3958643

[77] Lee I, Ouk Kim Y, Park S-C, Chun J. 2016. OrthoANI: an improved algorithm and software for calculating average nucleotide identity. Int J Syst Evol Microbiol 66:1100–1103. doi:10.1099/ijsem.0.000760. 26585518

[78] Moriya Y, Itoh M, Okuda S, Yoshizawa AC, Kanehisa M. 2007. KAAS: an automatic genome annotation and pathway reconstruction server. Nucleic Acids Res 35:W182–W185. doi:10.1093/nar/gkm321. 17526522 PMC1933193

[79] Saier MH, Jr, Reddy VS, Tsu BV, Ahmed MS, Li C, Moreno-Hagelsieb G. 2016. The Transporter Classification Database (TCDB): recent advances. Nucleic Acids Res 44:D372–D379. doi:10.1093/nar/gkv1103. 26546518 PMC4702804

[80] Wodke JAH, Puchałka J, Lluch‐Senar M, Marcos J, Yus E, Godinho M, Gutiérrez‐Gallego R, dos Santos VAPM, Serrano L, Klipp E, Maier T. 2013. Dissecting the energy metabolism in Mycoplasma pneumoniae through genome‐scale metabolic modeling. Mol Syst Biol 9:653. doi:10.1038/msb.2013.6. 23549481 PMC3658275

[81] Steffensen JL, Dufault-Thompson K, Zhang Y. 2016. PSAMM: a portable system for the analysis of metabolic models. PLoS Comput Biol 12:e1004732. doi:10.1371/journal.pcbi.1004732. 26828591 PMC4734835

